# Key Node Identification in Spatial Information Networks via Soft Community Partition and Hypergraph Interweaving Degree

**DOI:** 10.3390/s26185870

**Published:** 2026-09-16

**Authors:** Xiaolan Yu, Wei Xiong, Ping Jian, Yali Liu

**Affiliations:** Graduate School, Space Engineering University, Beijing 101416, China; yuxiaolan@hgd.edu.cn (X.Y.);

**Keywords:** spatial information networks, hypernetwork, soft community partition, interweaving degree, key node mining

## Abstract

**Highlights:**

This paper mainly adopts hypernetwork modeling to characterize the higher-order coupling of Spatial Information Networks, proposes a critical node identification algorithm based on KL NMF soft community partitioning and interweaving degree to achieve critical node identification under multi-service collaboration, and proposes a risk-level-based hierarchical protection strategy to reliably and comprehensively improve network resilience and reduce defense costs.

**What are the main findings?**

- A hypernetwork model of Spatial Information Networks is established.
- A key node identification algorithm for Spatial Information Networks based on soft community partitioning and interweaving degree is proposed.

**What are the implications of the main findings?**

- Establish a hypernetwork analysis model for Spatial Information Networks in multi-service scenarios.
- Accurately identify the critical nodes of large-scale and heterogeneous Spatial Information Networks.

**Abstract:**

Due to the widespread deployment of large-scale satellite constellations, Spatial Information Networks (SINs) exhibit increasingly high-order node and service coupling characteristics, rendering them vulnerable to node removal and cascading failures. Traditional graph-theoretic models fail to capture the inherent multi-node cooperative relationships within SINs, while conventional centrality metrics overlook service coupling features and cross-community propagation risks, leading to inaccurate key node identification. To address these issues, this paper proposes a key node mining method based on soft community partition and hypergraph interweaving degree. The method first constructs a hypernetwork to characterize the high-order associations among three types of services—communication, remote sensing, and navigation. Second, a probabilistic generative model, Hypergraph-MT, is introduced to detect overlapping communities and capture the multi-role attributes of satellites. Finally, the Interweaving Degree (IW) and its derived Bridge Score (IWBS) are defined to quantify node importance from two dimensions: load capacity and cross-community connectivity. Experimental results demonstrate that, compared with degree centrality and betweenness centrality, the proposed method increases the service interruption rate from 27.4% to 64.8% under a 5% removal ratio, and the service efficiency loss is elevated by more than 60%. In comparison with pure interweaving degree removal, IWBS triggers deeper cascading removal at a 30% removal ratio, reduces the removal cost by 8.9%, and improves the input–output ratio by 8.2% under high-intensity removal. Moreover, the algorithm exhibits linear scalability and strong robustness to parameter variations. The proposed method significantly enhances the accuracy and efficiency of key node identification in SINs, providing theoretical support for constellation deployment optimization and priority protection decision-making.

## 1. Introduction

Spatial Information Networks (SINs), as a core component of national critical infrastructure, are heterogeneous collaborative networks integrating multiple types of nodes, including low-, medium-, and high-orbit satellites, ground gateway stations, and aerial vehicles. Their primary mission is to provide diversified services such as global-coverage communication, navigation, remote sensing, and emergency support. In recent years, with the successive deployment and continuous expansion of large-scale constellation systems such as SpaceX’s Starlink and China’s Hongyan constellation, the network node scale has grown from hundreds to tens of thousands. Meanwhile, the coupling patterns between nodes and services exhibit significant high-order characteristics: a single node is required to simultaneously undertake multiple types of services, including communication relay, navigation enhancement, and remote sensing data distribution, while each service relies on the coordination of multiple nodes. This gives rise to a complex coupled network in which the node domain and the service domain are deeply intertwined. As the network scale increases by orders of magnitude and the high-order node–service coupling becomes increasingly intricate, the identification of critical nodes in SINs has emerged as a fundamental research need in system operation and management. On the one hand, the expanding network scale necessitates the use of critical node identification to support targeted operation and management practices. On the other hand, high-order coupling implies that node importance is no longer determined solely by topological location, but is closely tied to multi-service carrying capacity and cross-service collaborative contributions, rendering traditional topology-based evaluation metrics insufficient for characterizing such complex interdependencies. Consequently, a key challenge in this domain is how to effectively incorporate high-order coupling information into the identification of critical nodes within highly coupled SINs, which has increasingly become a research focus.

In recent years, certain progress has been made in the analysis of satellite network topology. In terms of network modeling, Refs. [1,2,3] have proposed combining hypernetwork theory with complex network topologies of satellite networks, characterizing the service logic layer of satellite networks from multiple perspectives and achieving mutual mapping between service scenarios and network topology. Therefore, in the face of ever-growing satellite network scales and user mission demands, incorporating hypernetworks to characterize satellite network topology and analyze their high-order network information has become a reliable and cutting-edge research focus. Regarding key node identification, traditional methods such as degree centrality [4] and betweenness centrality [5]—as well as their modifications—remain central to screening important nodes in satellite network topology. However, as satellite network scales continue to expand, the identification of important nodes has gradually shifted from microscopic to mesoscopic and macroscopic levels. Recent studies [6,7,8] have explored community detection methods for key node identification in satellite network topologies. In the field of high-order network analysis, Battiston et al. [9] systematically elaborated on research progress beyond pairwise interactions, pointing out the limitations of traditional pairwise interaction models. Contisciani et al. [10] proposed inference methods for hyperedges and overlapping communities in hypernetworks, providing technical support for high-order network community detection. Chodrow et al. [11] developed generative hypergraph clustering methods, enriching the technical system for hypernetwork-based community analysis. Furthermore, high-order network analysis theory has been successfully applied to complex network analysis in domains involving numerous and intricately interacting elements, such as transportation, traffic, and trade [12,13,14]. Especially with the development of UAV swarms, which have been widely used in complex network analysis, network analysis methods have also been introduced to analyze the information transmission process of communication and control in the field of robot teams [15].

Despite existing multifaceted research on satellite networks, significant bottlenecks and deficiencies remain in adapting to the high-order coupling characteristics of large-scale SINs, given the rapidly increasing network scale and diverse mission requirements. These deficiencies are mainly reflected in four aspects. First, the challenge of characterizing high-order associations: traditional network models can only describe pairwise node-to-node interactions and cannot effectively represent the high-order relationships of multi-node collaborative services. Consequently, the coupling features between services and nodes are overlooked, leading to intrinsic biases in network structure analysis. Second, the limitation of key node identification: conventional centrality metrics only consider the number of topological connections or the betweenness role of a node, ignoring its service-carrying capacity and cross-service coupling degree. This may misclassify nodes with “high connectivity but low service value” as key nodes while missing core hubs that span multiple services and communities. Third, the lack of overlapping community detection capabilities: nodes in SINs generally possess multi-role attributes (e.g., navigation, communication, and remote sensing). However, traditional algorithms such as Louvain [16] and projection-based methods [17] only support hard partitioning where each node belongs to a single community, failing to capture the mixed-membership characteristics of nodes, resulting in community structure analyses that deviate from actual network operation.

To address the above issues, this study proposes a key node mining method for SINs based on soft community partition and hypergraph interweaving degree. The specific approach is as follows. First, breaking through the limitations of traditional models, a hypernetwork structure is introduced to accurately characterize the high-order associations between nodes and services in SINs. Second, based on the hypernetwork characteristics, an interweaving degree (IW) metric is proposed, integrating three layers of information—topological connections, service-carrying capacity, and cross-service degree—thereby overcoming the limitation that conventional centrality indices focus on topological structure while paying insufficient attention to service demands. Third, a probabilistic generative model, Hypergraph-MT, is introduced to achieve overlapping community detection, accurately capturing the multi-role attributes of nodes and filling the gap left by traditional algorithms that cannot handle mixed-membership communities.

This study aims to provide a novel methodological framework for high-order structural analysis and key node mining in large-scale SINs. The proposed method is expected to accurately identify core hub nodes and vulnerable nodes in the network, offering direct guidance for engineering applications such as constellation deployment optimization and prioritization of protection measures. The remainder of this paper is organized as follows. Section 2 presents an overview of SINs and related work. Section 3 establishes the methodological framework for key node mining in SINs. Section 4 designs simulation experiments to demonstrate the reliability and superiority of the proposed method. Section 5 concludes the paper and discusses directions for future research.

## 2. Overview of Key Node Mining in Spatial Information Networks

As integrated space–ground heterogeneous collaborative networks, Spatial Information Networks (SINs) consist of a satellite network layer and a ground user layer. With the integration of communication, navigation, and remote sensing as their core development trend, SINs provide diversified services including global communication, navigation, remote sensing, and emergency support [18]. To comprehensively analyze the novelty and necessity of the proposed method, this section provides an overview of key node mining in SINs from two aspects. First, the composition and structure of the target object—SINs—are summarized and analyzed to provide a basis for selecting key node mining methods. Second, the definition of key node mining in SINs is given, and the progress of high-order network analysis methods applied to SINs, satellite networks, and related fields is reviewed and analyzed. Finally, the feasibility of the proposed method is discussed.

### 2.1. Spatial Information Networks

**Figure 1:**
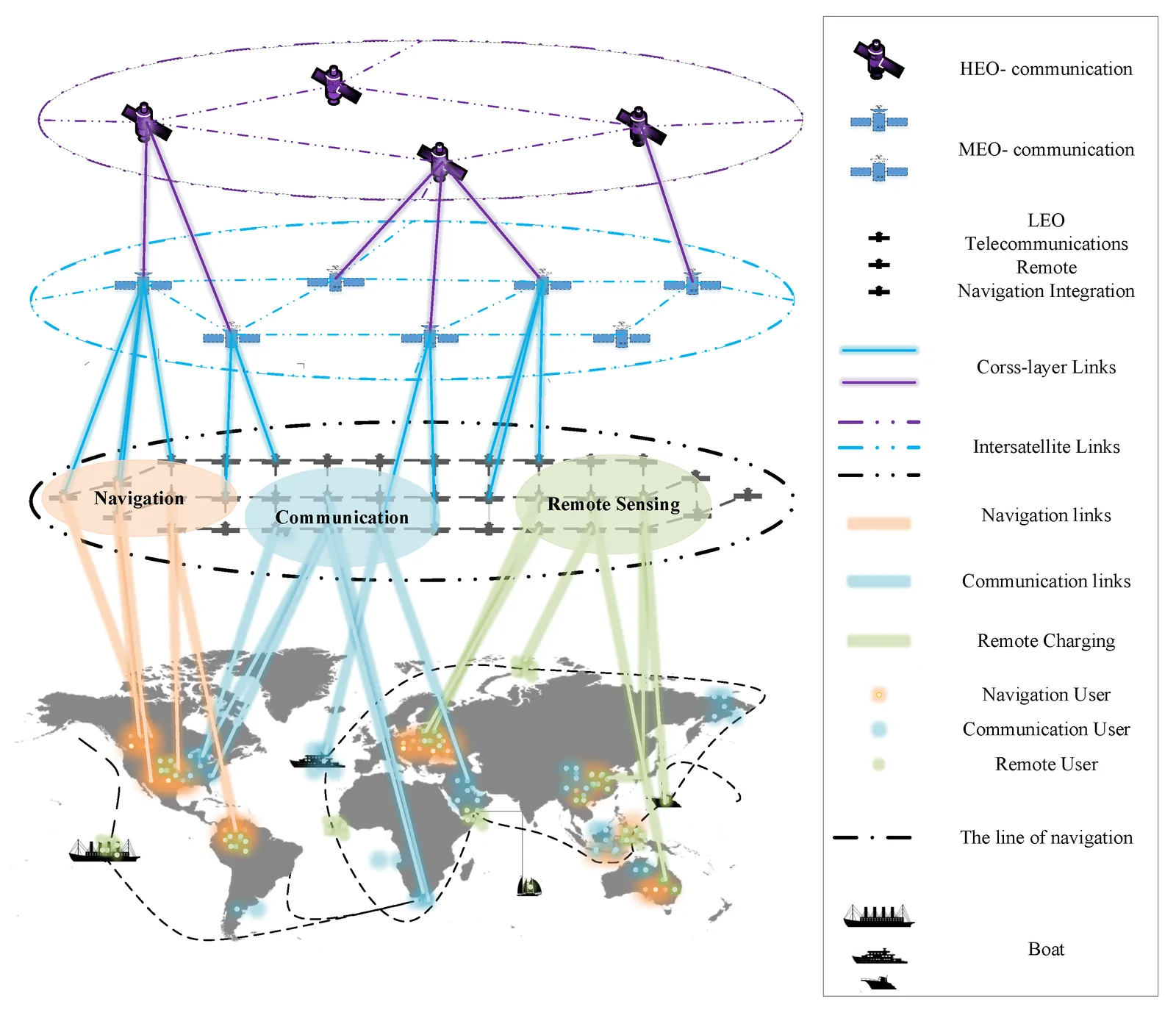
Composition Architecture of Spatial Information Networks.

The overall architecture of SINs exhibits typical characteristics of multi-level hierarchy, cross-domain coverage, high dynamics, and strong coupling. It can be divided into two core levels: the satellite network layer in the space domain and the ground user layer in the land and sea domains. Each level has significantly different functional characteristics and operational patterns, yet they interact and are deeply coupled, jointly determining the service capability and operational performance of the network. To intuitively present the overall architecture and hierarchical logic of SINs, this paper constructs a structural diagram of the architecture, as shown in Figure 1.

(1) Satellite Network Layer: The satellite network layer adopts a hybrid constellation architecture dominated by low-orbit satellites supplemented by medium- and high-orbit satellites. Low Earth Orbit (LEO) satellites serve as the core platform for the integration of communication, navigation, and remote sensing (CNR). Represented by constellations such as Starlink, OneWeb, and China Satellite Network, LEO satellites operate at orbital altitudes mainly between 500 km and 1500 km. They offer advantages such as short propagation delay, low link loss, and large-scale deployment, and undertake core services including communication relay, remote sensing observation, and navigation enhancement, forming multi-service carrying node clusters. Medium Earth Orbit (MEO) satellites (altitudes between 2000 km and 20,000 km) and High Earth Orbit (HEO) satellites (altitude 35,786 km) serve as functional supplements to alleviate the communication load of LEO constellations. The integration of CNR functions requires a single LEO satellite to simultaneously carry multiple services, while each service relies on the coordinated operation of several satellites. This fully demonstrates that the satellite network layer exhibits complex coupling relationships between nodes and services, imposing higher demands on network service resilience and dynamic reconfiguration capabilities [18].

(2) Ground User Layer: Based on the functional diversity of SINs, the ground user layer forms three core user groups: communication, remote sensing observation, and navigation. The distribution of these users shows significant heterogeneity and imbalance, which directly affects network load distribution and resource scheduling strategies. Communication users are mainly concentrated in densely populated developed regions, focusing on real-time communication needs of personal terminals and ground stations. Remote sensing observation users include land-based critical area nodes and ocean observation nodes along important shipping routes, as well as ground data reception and processing stations, serving scenarios such as resource exploration and disaster monitoring. Navigation users cover various mobile terminals on both oceans and land, meeting high-frequency demands in densely populated areas while also ensuring navigation support in remote regions [19]. This uneven distribution characteristic makes the service demands of SINs exhibit regional aggregation and dynamic fluctuation patterns.

(3) Service Layer: In SINs, the service layer serves as the functional bridge between the satellite infrastructure and ground user demands, encompassing three primary service types: communication relay, navigation enhancement, and remote sensing data distribution. Unlike conventional single-service networks, these services in SINs are not isolated but are jointly fulfilled through the collaborative operation of multiple satellite nodes across different orbital altitudes. Meanwhile, the heterogeneous and unbalanced distribution of ground users introduces regional aggregation effects, which further impose dynamic and non-uniform service load patterns on the satellite network layer. The interplay between the multi-service coupling within the satellite layer and the spatio-temporal heterogeneity of ground user demands gives rise to strongly coupled and multi-constrained operational characteristics. Consequently, network performance depends not only on the topological positions of satellite nodes, but also on their multi-service carrying capabilities and cross-community connectivity strengths.

Based on the above analysis, to fully account for the node–service coupling relationships, regional aggregation of user demands, and cross-service coordination capacity in SINs, this paper adopts hypernetwork modeling to accurately characterize the high-order associations between network nodes and service demands. Subsequently, theoretical methods such as hypergraph interweaving degree and overlapping community detection are introduced to provide a theoretical basis for key node mining algorithms.

### 2.2. Key Node Mining in Spatial Information Networks

**Figure 2:**
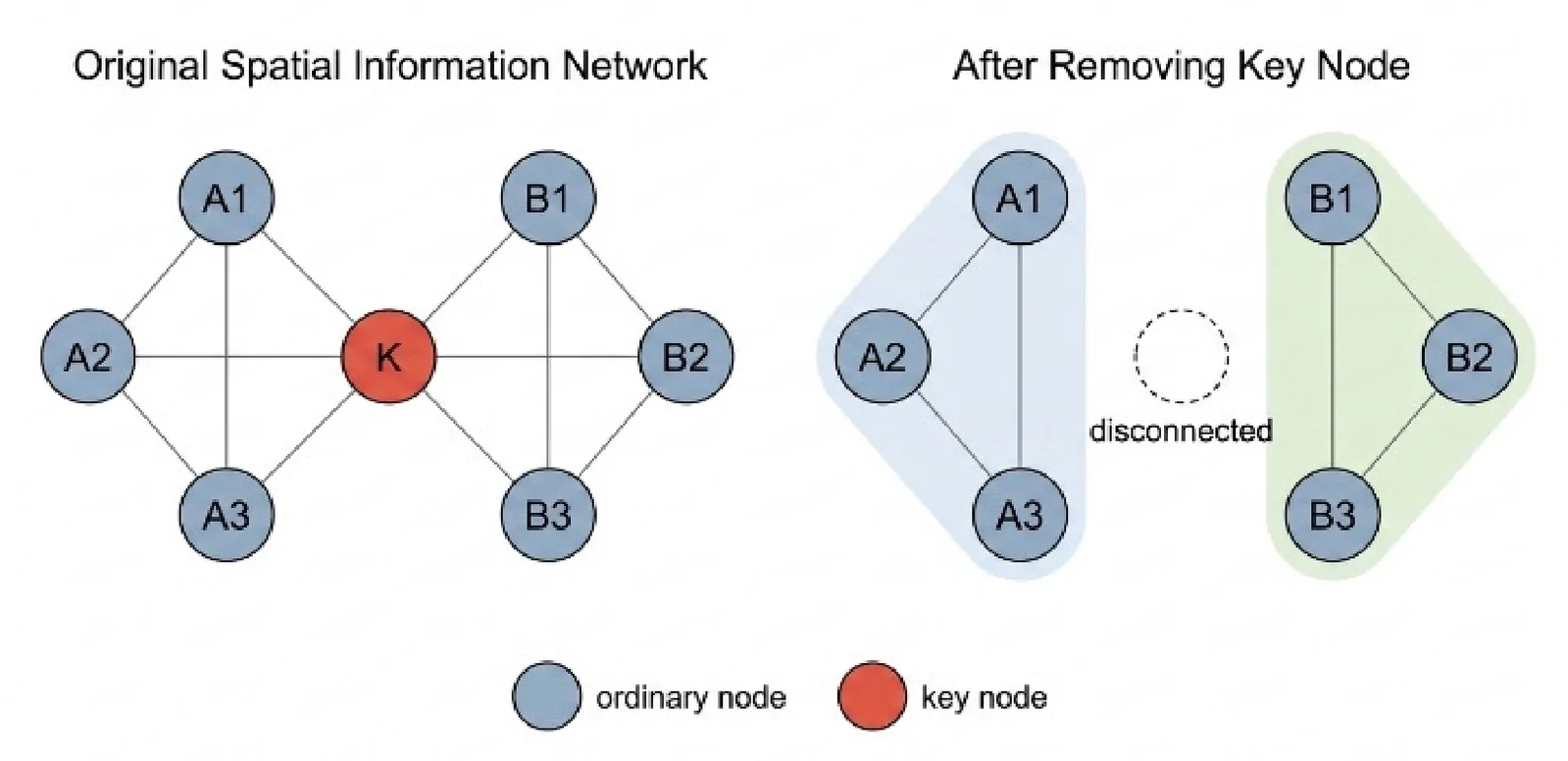
Key Node Mining.

As a highly dynamic complex network system, SINs involve multi-layer interactions among satellites, ground stations, and user terminals. Key node mining techniques in SINs aim to identify nodes that significantly affect network connectivity, robustness, and service performance. These nodes typically include high-load satellites or critical relay stations, whose removal may trigger cascading failures or service interruptions, as shown in Figure 2. In recent years, with the rapid development of satellite constellations (e.g., Starlink), key node mining has become a core research direction for enhancing the resilience of SINs. Nevertheless, existing methods still face challenges such as multi-service heterogeneous interactions and resource constraints. This section discusses current related work on the topology analysis of SINs. It first focuses on network model construction, particularly the application of hypernetwork theory to capture multi-service and multi-node interactions in SINs. Second, it discusses extensions of traditional methods, such as centrality analysis, hypernetwork-specific metrics, and the role of community detection methods in key node identification. Finally, it analyzes the limitations of these methods and points out future directions.

(1) Model Construction

The topological structure of SINs differs from that of traditional single-layer networks. Their multi-service nature leads to high-order, multimodal interactions among nodes. Traditional graph models struggle to capture these complex relationships; consequently, hypernetwork theory has become a mainstream approach for constructing SIN models. Hypernetworks allow a single hyperedge to connect multiple nodes, perfectly simulating scenarios in SINs where multiple satellites collaborate to perform a service. Model construction for SINs mainly focuses on three aspects: traditional network topology, hierarchical network topology, and time-varying dynamic network topology.

Traditional network topology: Xiao et al. established a conventional satellite network topology using simulation and computation, employing the graph-theoretic concepts of nodes and edges to describe the interaction model between satellites [20].

Hierarchical network topology: Guo et al. and Kawamoto et al. used hierarchical network models to describe the composition of satellite networks [21,22], enabling more accurate characterization of satellite network structural properties. To describe large-scale satellite clusters more precisely, Li et al. proposed a K-layer satellite network model, which flexibly represents satellite network topologies and provides a more comprehensive framework for network model construction [23].

Time-varying dynamic network topology: Refs. [24,25,26] all adopt dynamic time-varying network models to construct satellite network models, primarily for resource management and policy optimization in on-orbit dynamic routing and network communication.

Hypernetwork: as satellite scales continue to expand and functions become increasingly rich, to cope with growing scalability and heterogeneity, some researchers have recently extended hypernetworks to multi-layer frameworks of SINs to handle node–service heterogeneity. In view of the multi-node cooperation characteristics of SINs, Ref. [3] proposed using hypernetwork theory to abstract SIN services as hyperedges, established a single-layer SIN model, and presented a resilience analysis framework for SINs, effectively solving the problem of resilience/elasticity analysis models for SINs in multi-service scenarios. Building on this, Ref. [27] proposed a vulnerability analysis model for SINs based on multi-layer hypernetworks, established a multi-layer hypernetwork structure, and accurately characterized the mapping between uniformly distributed satellite networks and unevenly distributed user networks. Based on hypernetwork metrics, a key node mining strategy using hypernetwork overlap degree was proposed to accurately identify key nodes in multi-service scenarios. Therefore, in multi-service scenarios, hypernetwork theory captures high-order interactions among satellites and improves model sensitivity to cascading failures. Overall, hypernetwork theory provides a solid foundation for key node mining in SINs.

(2) Key Node Identification

Research on key node mining mainly proceeds from two perspectives: traditional methods (e.g., centrality measures) and community detection methods (e.g., modularity optimization and community detection algorithms), and their potential applications in SINs are discussed.

First, traditional methods are mainly based on network topology and node attributes, identifying key nodes using metrics such as degree centrality, betweenness centrality, and closeness centrality. These methods assume that the network structure is relatively stable and are suitable for static or quasi-static scenarios. In SINs, such methods are often used to identify backbone nodes in satellite networks to optimize routing and load balancing. Early work by Freeman (1977) [28] laid the theoretical foundation of centrality. However, as the scale of satellite networks continues to grow, traditional centrality and betweenness centrality methods are no longer sufficient to accommodate the ever-increasing scale and functional attributes of SINs. To address the dynamics of SINs, Refs. [29,30,31] decompose network dynamics using dynamic time-varying graph methods and employ convolutional neural networks and deep reinforcement learning to improve the efficiency of key node mining and meet the demands of large-scale, highly dynamic network key node identification. Nevertheless, these studies mainly focus on communication services and single-layer large constellations. As the scale of SINs expands, their functional attributes become richer. To solve this problem, Ref. [32] proposed a mission-oriented personalized network analysis method, establishing a network architecture for a four-layer (ground–water–air–space) SIN and fully considering the hierarchical division and mission attributes of SINs. Ref. [33] introduced coverage centrality metrics and hypernetwork theory to study the robustness of SINs. Furthermore, Refs. [32,34,35] indicate that traditional graph theory-based key node mining methods have been widely applied to aspects such as robustness, survivability, and load balancing in SINs.

As the scale of SINs continues to increase, key node mining should not be limited to microscopic node importance analysis. Based on complex network theory, from a macroscopic perspective, techniques such as community detection have also found many applications. For example, Ref. [36] proposed a relative important node mining algorithm based on community detection and biased random walk with restart (CDBRWR) to address these issues. This method integrates network community information into the mining of relatively important nodes for the first time and introduces a novel biased random walk strategy, achieving accurate and efficient mining of relatively important nodes in various networks. Ref. [37] evaluated key nodes in social networks using community detection-based bridge nodes. Therefore, community detection, as a method that can explain the macroscopic properties of complex networks, can effectively mine key nodes in complex networks. This provides a cutting-edge approach for key node mining in SINs.

### 2.3. Summary

In summary, this section has systematically analyzed the architectural characteristics of SINs and the research progress of key node mining methods, thereby fully demonstrating the novelty and necessity of the method proposed in this paper. As an integrated space-ground heterogeneous collaborative network, SINs consist of a satellite network layer and a ground user layer, with the integration of CNR as their core development trend. They exhibit significant multi-level, heterogeneous, and strongly coupled characteristics. There is, therefore, an urgent need for more targeted theoretical approaches to characterize the high-order association features of service-oriented SIN topologies and to mine key nodes comprehensively and accurately.

Regarding key node mining methods for SINs, current approaches have certain limitations. On the one hand, they ignore the mapping relationship between service demands and network topology. On the other hand, they fail to adequately accommodate the dynamics and heterogeneity of SINs, making it difficult to meet the network analysis requirements in CNR-integrated scenarios. Therefore, through a systematic analysis of SIN architectural characteristics, this section clarifies the adaptation requirements for theoretical methods. Meanwhile, by reviewing relevant research progress and identifying the shortcomings of existing methods, it further highlights that this paper proposes, on the basis of hypernetwork models, a key node mining algorithm for SINs under high-order network conditions. This work provides research references and application suggestions for SIN structure optimization and service robustness enhancement.

## 3. Key Node Mining Model for Spatial Information Networks Based on Soft Community Partition and Hypergraph Interweaving Degree

This section aims to propose a key node mining model for SINs, which are characterized by service diversity and topological complexity.

**Figure 3:**
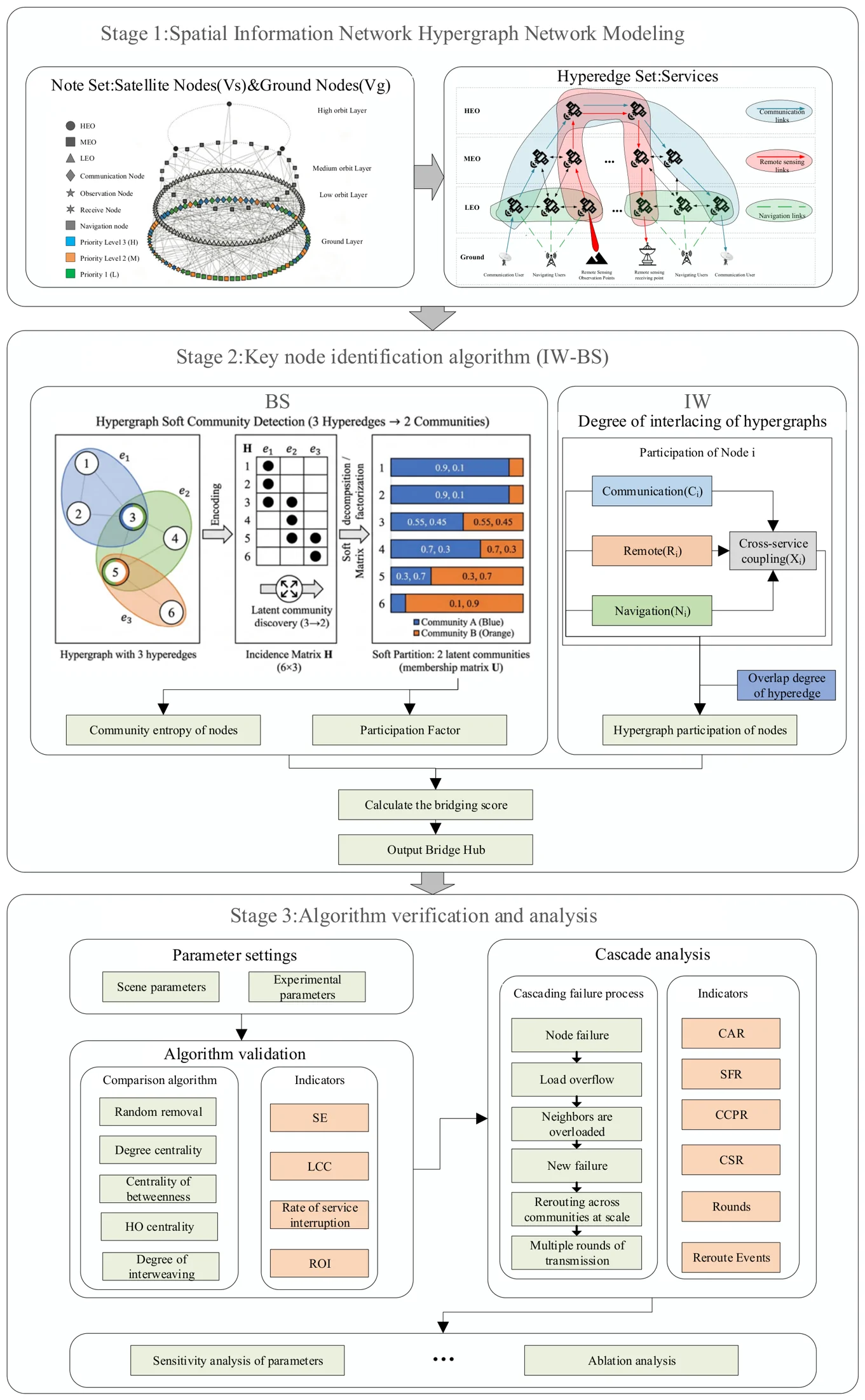
Key Node Mining Model for Spatial Information Networks.

As shown in Figure 3, the proposed model mainly consists of three core stages. The first stage is SIN model construction, which aims to characterize the multi-node to multi-service mapping relationship based on hypernetwork theory, thereby establishing a node-service SIN model. The second stage is the key node mining algorithm, which aims to accurately, effectively, and reliably identify key nodes in the multi-node, multi-service network model based on soft community partition and hypergraph interweaving degree theory. The third stage is algorithm verification and analysis, which aims to validate the rationality and superiority of the proposed method through structural performance metrics and cascading propagation metrics.

### 3.1. Hypernetwork Model of Spatial Information Networks

This subsection first defines the boundaries of the research subject and constructs the model, laying the foundation for subsequent theoretical analysis and simulation experiments. It is clarified that an SIN is a complex system consisting of space nodes, ground nodes, and various services jointly supported by these nodes. However, traditional graph models use edges to represent pairwise relationships between nodes, which makes it difficult to characterize the group property of multi-node collaboration to accomplish a single service in satellite networks. To accurately characterize the topological structure and functional dependencies, we abstract the SIN as a heterogeneous hypernetwork model [26]. Reference [26] defines a hypernetwork as H=(V,E), where V is the set of nodes and E is a set of hyperedges, each hyperedge e∈E being a non-empty subset of V that can contain any number of nodes. Therefore, for the SIN proposed in this paper, the node set and hyperedge set are defined as follows:

The node set V represents the physical entities in the network, which consists of two types of heterogeneous nodes.

The satellite node set ($V_{s}$) refers to various satellites in orbit, such as communication satellites, navigation satellites, and remote sensing satellites. They are the core components of the network, undertaking key functions such as data relay, signal broadcasting, and information acquisition.

The ground node set ($V_{g}$) refers to various ground facilities and users on the Earth’s surface, such as TT&C stations, data receiving stations, injection stations, observed objects, and ground navigation/communication users. Satellite nodes can provide them with services such as communication, remote sensing, and navigation. Therefore, the node set is defined as $V=V_{s}\cup V_{g}$.

The hyperedge set E represents the various services provided by the network, where each hyperedge is a subset of the node set V.

The communication service $h_{com}\in E$ consists of at least two ground nodes and one satellite node, $h_{com}=\{g_{i},s_{m},g_{j}\}$, where $\{g_{i},g_{j}\}\in V_{g}$ are ground communication users, and $\{s_{m}\}\in V_{s}$ is a satellite node equipped with communication payloads.

The remote sensing service $h_{re}\in E$ consists of at least two ground nodes and one satellite node, $h_{re}=\{g_{i},s_{m},g_{j}\}$, where $\{g_{i},g_{j}\}\in V_{g}$ are ground observation nodes and remote sensing data receiving nodes, $\{s_{m}\}\in V_{s}$ is a satellite node equipped with remote sensing payloads.

The navigation and positioning service requires at least three navigation satellites $\left\{s_{1},\;s_{2},\;s_{3},\;s_{4}\right\}\in V_{s}$ and at least one ground user $\left\{g_{1}\right\}\;\in \;V_{g}$ to jointly form a service hyperedge. Therefore, this hyperedge can be expressed as $h_{nav}\;=\left\{s_{1},\;s_{2},\;s_{3},\;s_{4},\;g_{1}\right\}$.

In summary, the boundary of the SIN studied in this paper is strictly defined as the physical entity set consisting of satellite nodes and ground nodes, together with the functional set formed by the service hyperedges (communication, navigation, remote sensing, etc.) that they collaboratively support.

### 3.2. Key Node Mining Algorithm Based on Soft Community Partition and Hypernetwork Interweaving Degree

To analyze SINs from a macroscopic perspective, this subsection first abstracts the topology of the spatial information hypernetwork (which contains service information) into a hypernetwork incidence matrix, thereby further elevating node and service information into high-order network information. Then, a community partition method is adopted to divide satellite nodes according to the distribution of service hyperedges. Since satellite nodes in SINs participate in multiple service hyperedges to varying degrees, the nodes do not strictly belong to a single community when performing community partition. To capture this overlap and fuzziness, this paper employs the Kullback–Leibler divergence-based non-negative matrix factorization (KL-NMF) method [10] to perform a soft partition of the SIN hypernetwork model.

Subsequently, to accurately characterize the interweaving of different services (communication, navigation, remote sensing, etc.) in SINs, this paper constructs the Interweaving Degree (IW) metric. Based on communication hyperedges, remote sensing hyperedges, and navigation hyperedges, the IW metric statistically accounts for node service participation, hyperedge overlap, and cross-service interweaving terms, thereby characterizing the activity of nodes in multi-service structures.

Finally, this paper integrates the IW with the soft community partition results to form a Bridge Score (BS). Combined with a screening mechanism based on high entropy and high interweaving degree, the proposed method identifies cross-service, cross-community bridging hubs, denoted as IWBS. This approach provides a finer-grained structural characterization and lays the foundation for subsequent key node identification and analysis in SINs.

#### 3.2.1. Definitions of Soft Community Partition and Interweaving Degree

1. Hypernetwork incidence matrix

Based on the model assumptions in Section 3.1, let the node set in the satellite network be $V=\{v_{1},v_{2},\cdots ,v_{n}\}$ and the hyperedge set be $E=\{e_{1},e_{2},\cdots ,e_{m}\}$, where each hyperedge represents a set of satellite nodes jointly participating in a certain service. To convert the hypernetwork structure into a computable form, we construct a node–hyperedge incidence matrix:

(1) $$H={\mathbb{R}}^{n\times m}$$

with its elements defined as:

(2) $$H_{ij}=\left\{\begin{array}{l} 1,v_{i}\in e_{j}\\ 0,v_{i}\notin e_{j} \end{array}\right.$$

Each row of this matrix corresponds to the hyperedge participation of a node, and each column corresponds to the node composition of a hyperedge. Since services in hypernetworks often have multi-node collaboration properties, unlike the adjacency matrix in ordinary graphs, the incidence matrix can more accurately reflect the service-layer structure.

2. KL-NMF decomposition model

To mine the latent community structure in the hypernetwork, this paper decomposes the incidence matrix H into the product of two non-negative matrices:

(3) $$H\approx UV^{T}$$

where

$U\in {\mathbb{R}}_{+}^{n\times k}$ is the node–community membership matrix;

$V\in {\mathbb{R}}_{+}^{n\times m}$ is the hyperedge–community weight matrix;

k is the preset number of communities.

The i-th row of $U_{i}=(u_{i1},u_{i2},\cdots ,u_{iK})$, can be interpreted as the membership strength of node $v_{i}$ to each community. If each row is normalized, it can be regarded as a probability distribution:

(4) $$p_{ik}=\frac{u_{ik}}{\sum_{t=1}^{K}u_{it}},\sum_{k=1}^{K}\begin{array}{l} p_{ik}=1 \end{array}$$

Therefore, nodes are not strictly assigned to a single community but may belong to multiple communities simultaneously in a probabilistic manner. This soft partition approach is more suitable for the two-layer hypernetwork model of SINs, which exhibits overlapping structures.

3. Definition of Interweaving Degree (IW)

Since SINs are primarily concerned with the coupling degree of nodes in communication, remote sensing, and navigation services, certain nodes in the network not only undertake multiple services but also participate in several different types of hyperedges simultaneously. Such nodes usually possess high structural importance. To better capture this characteristic, this subsection defines the Interweaving Degree (IW) of a node to measure its service coupling strength in the hypernetwork. The IW not only reflects the number of services a node participates in but also indicates the degree of correlation among different services. Therefore, compared with pure degree centrality or hyperedge participation count, the IW better characterizes the functional complexity of nodes.

Let the participation degree of node $v_{i}$ in the communication service hypernetwork be $C_{i}$, in the remote sensing service hypernetwork be $R_{i}$, and in the navigation service hypernetwork be $N_{i}$; the hyperedge overlap degree be $O_{i}$; and the cross-service coupling term be $X_{i}$. The cross-service coupling term is defined as:

(5) $$X_{i}=\sqrt{C_{i}R_{i}N_{i}}$$

Then, the Interweaving Degree of a node is defined as:

(6) $$IW_{i}=\alpha C_{i}+\beta R_{i}+\gamma N_{i}+w_{o}O_{i}+w_{x}X_{i}$$

where

α is the weight of communication services;

β is the weight of remote sensing services;

γ is the weight of navigation services;

$w_{o}$ is the weight of the overlap term;

$w_{x}$ is the weight of the cross-service coupling term.

Furthermore, α+β+γ=1, which balances the importance of the communication layer, remote sensing layer, and navigation layer. The term $X_{i}$ comprehensively considers the node’s participation degree in the three types of services and their coupling relationships.

From a structural perspective, nodes with a high Interweaving Degree typically exhibit the following characteristics: (i) they participate in multiple communication, remote sensing, and navigation services; (ii) they are located at the intersection of multiple hyperedges; (iii) they possess strong carrying and relaying capabilities in the local network; and (iv) they have a significant impact on load redistribution and path reconfiguration. Therefore, the Interweaving Degree can be regarded as a kind of “service coupling centrality,” emphasizing the local importance of nodes in multi-service collaboration.

However, a high Interweaving Degree alone does not necessarily imply that a node possesses cross-community propagation capability. If a node participates in many services but belongs to a single community, its influence may be confined to the local structure. Therefore, it is necessary to further combine community coupling and the participation coefficient to identify truly bridging hubs.

#### 3.2.2. Soft Community Partition Algorithm for Hypernetworks Based on KL-NMF

1. Objective Function and Optimization Problem

The core objective of KL-NMF is to minimize the KL divergence between the original matrix H and the reconstructed matrix $UV^{T}$. The objective function is defined as:

(7) $$\underset{U,V}{\min}D_{KL}(H||UV^{T})$$

where

(8) $$D_{KL}(H||UV^{T})=\sum_{i=1}^{n}\sum_{j=1}^{m}\left[H_{ij}\log\frac{H_{ij}}{{\left(UV^{T}\right)}_{ij}}-H_{ij}+{\left(UV^{T}\right)}_{ij}\right]$$

In the above, both U and V are required to be element-wise non-negative, which is consistent with the semantics of node–community relationships and hyperedge–community contributions. The KL divergence has good modeling capability for sparse non-negative matrices.

2. Multiplicative Update Rules

To solve the optimization problem of the objective function, this subsection adopts an alternating multiplicative update strategy to iteratively update U and V. Let the current reconstructed matrix be:

(9) $$\overset{\wedge }{H}=UV^{T}$$

Then, according to the update rules of KL-NMF, we obtain:

(10) $$\begin{array}{l} U\leftarrow U\Theta \frac{(H/\overset{\wedge }{H})V}{1V}\\ V\leftarrow V\Theta \frac{{\left(H/\overset{\wedge }{H}\right)}^{T}U}{1U} \end{array}$$

where

Θ denotes element-wise multiplication;

$H/\overset{\wedge }{H}$ denotes element-wise division; 

1 denotes an all-one matrix or the summation term used for normalization.

The above update process has two notable advantages: first, the updated matrices remain non-negative; second, the iterative form is simple and stable, making it suitable for handling large-scale sparse incidence matrices. In practice, randomly initialized U and V can be used as starting values, and the updates are performed alternately until convergence or the maximum number of iterations is reached.

3. Node Community Probabilities and Dominant Community Identification

After solving for U through iterative updates, the node–community probability matrix U is obtained. For a node $v_{i}$, its normalized community probability vector is:

(11) $$P_{i}=(p_{i1},p_{i2},\cdots ,p_{iK})$$

where $p_{ik}$ denotes the probability or membership strength that node $v_{i}$ belongs to the k-th community. Furthermore, the dominant community of a node can be defined as:

(12) $$c_{i}^{*}=\arg\underset{k\in (1,\cdots ,K)}{\max}p_{ik}$$

This expression gives the strongest community affiliation of the node in a probabilistic sense, but it does not treat it as the exclusive affiliation. Instead, the multi-community participation property is retained for subsequent analysis of cross-community behaviors.

4. Definition of Community Entropy

To further characterize the dispersion degree of a node’s community affiliations, this subsection defines the community entropy as:

(13) $$Entropy(i)=-\sum_{k=1}^{K}p_{ik}\log(p_{ik}+\varepsilon )$$

where s is a small constant to avoid taking the logarithm of zero.

Community entropy reflects the uniformity of a node’s distribution across multiple communities. If a node belongs almost exclusively to a single community, its entropy is low; if a node has strong membership in multiple communities, its entropy is high. A higher entropy indicates that the node’s structural role is more ambiguous and that the node is more likely to lie at community boundaries or in cross-community connecting positions.

5. Definition of Participation Coefficient

In addition to entropy, this subsection further introduces the participation coefficient to measure the degree of a node’s cross-community involvement. It is defined as:

(14) $$PC(i)=1-\sum_{k=1}^{K}p_{ik}^{2}$$

This metric characterizes the dispersion of a node’s community membership. If a node belongs almost entirely to a single community, PC(i) is small; if the node is more evenly distributed across multiple communities, PC(i) is large. Compared with entropy, the participation coefficient emphasizes the breadth of distribution, whereas entropy emphasizes the uncertainty of distribution. The combined use of both provides a more comprehensive description of a node’s cross-community attributes.

Thus, through soft community partition of the hypernetwork via KL-NMF, we obtain probabilistic membership of nodes in multiple communities. This result can be used not only to identify a node’s dominant community but also to further compute community entropy and participation coefficient, laying the foundation for subsequent interweaving degree and bridging hub identification algorithms. The pseudocode for Algorithm 1 looks like this.
**Algorithm 1** KL-NMF-Based Soft Community Detection for HypergraphsInput: incidence matrix H, number of communities K, maximum number of iterations T, convergence threshold ε
Output: node-community matrix U, hyperedge-community matrix V, node soft membership matrix P, dominant community labels $C^{*}$, community entropy Entropy, participation coefficient PC
1:    Randomly initialize nonnegative matrices U and V, and normalize each row of U
2:    Set the current loss $D_{prev}=+\infty$
3:    for t = 1 to T do4:            Compute the reconstruction matrix $A=UV^{T}$
5:            Compute the KL divergence loss D between H and A
6:            if $|D-D_{prev}|<\varepsilon$ then7:                    break8:            end if9:            Update U and V to gradually reduce the KL divergence10:          Renormalize each row of U
11:          Set $D_{prev}=D$
12:  end for13:    Normalize U to obtain the node soft membership matrix P
14:    Compute node community entropy Entropy based on P
15:    Compute participation coefficient PC based on P
16:    Return U, V, P, Entropy, and PC


#### 3.2.3. Key Node Mining Algorithm Based on Interweaving Degree Bridge Score (IWBS)

1. Definition of IWBS

The key node mining algorithm based on IWBS can identify key nodes that simultaneously possess a high interweaving degree, strong cross-community participation capability, and high ambiguity in community membership. Once such nodes are removed, they not only affect local service execution but may also trigger wider cascading propagation through cross-community paths. To quantify this characteristic, this paper constructs a comprehensive bridge hub score $IWBS_{i}$ defined as:

(15) $$IWBS_{i}=IW_{i}\cdot (1+\lambda_{1}PC(i))\cdot (1+\lambda_{2}Entropy(i))$$

where

$IW_{i}$ is the interweaving degree of node i;

PC(i) is the participation coefficient;

Entropy(i) is the community entropy;

$\lambda_{1},\lambda_{2}$ are tuning parameters.

In the formula, $IWBS_{i}$ reflects the service coupling strength of a node, PC(i) reflects the breadth of its cross-community participation, and Entropy(i) reflects the fuzziness of its community boundaries. Together, these three components determine whether a node possesses bridge hub attributes.

From a physical perspective, bridge hub nodes with high IWBS typically lie between multiple service clusters or community structures and serve as critical channels for information, load, and path reconfiguration. The removal of such nodes often triggers the following chain reaction: first, local service interruption; second, blockage of cross-community paths; third, load redistribution to other regions; and finally, overload and cascading removal of secondary nodes, leading to broader functional degradation.

Therefore, high-IWBS bridge hub nodes are different from ordinary high-centrality nodes. The latter mostly reflect the number of connections or the frequency of path occurrences, whereas bridge hub nodes emphasize the “cross-service, cross-community, cross-function” propagation channel attribute, which endows them with stronger structural influence.

2. Ranking of Key Nodes Based on IWBS

To facilitate comparison among different nodes, IWBS can be normalized. Let a given indicator vector be:

$X=\{x_{1},x_{2},\cdots ,x_{n}\}$ Then its normalized form can be expressed as:

(16) $${\tilde{x}}_{i}=\frac{x_{i}-\min(X)}{\max(X)-\min(X)}$$

After normalization, nodes can be sorted according to their $IWBS_{i}$ values, and the top-ranked nodes can be selected as the candidate set of bridge hubs. The pseudocode of the key node mining algorithm based on IWBS is shown below.

Algorithm 2 first defines the Interweaving Degree (IW) to characterize the coupling strength of nodes in communication, remote sensing, and navigation services. Then, based on the IW, it integrates the community participation coefficient and community entropy to construct the IWBS metric. This metric can simultaneously reflect both the local service importance and the global cross-community propagation capability of a node. Using this algorithm, key nodes with high risk propagation attributes in SINs can be identified more accurately.
**Algorithm 2** Key node mining algorithm based on IWBSInput: Soft community matrix P, community entropy Entropy, participation coefficient PC, communication hyperedge set $E_{c}$, Remote sensing hyperedge set $E_{r}$, navigation hyperedge set $E_{n}$, threshold θ, selection ratio r, and weight parameters $\alpha ,\beta ,\gamma ,w_{o},w_{x},\lambda_{1},\lambda_{2}$.Output: bridge hub set B, interweaving score IW, bridge score IWBS1:    Count the number of communication hyperedges incident to each node, denoted by C, and the number of navigation hyperedges, denoted by N, and the number of Remote sensing hyperedges, denoted by R2:    Compute the overlap among the hyperedges incident to each node, denoted by O3:    Compute the cross-service interweaving term $X_{i}=\sqrt{C_{i}R_{i}N_{i}}$
4:    Normalize C, N,R, and O
5:    Construct the interweaving score $IW_{i}=\alpha C_{i}+\beta R_{i}+\gamma N_{i}+w_{o}O_{i}+w_{x}X_{i}$
6:    Normalize PC and Entropy7:    Construct the bridge score $IWBS_{i}=IW_{i}\cdot (1+\lambda_{1}PC(i))\cdot (1+\lambda_{2}Entropy(i))$
8:    Rank nodes by interweaving score I and select the top r proportion as candidates9:    Among the candidates, keep nodes with Entropy(i)≥θ
10:  Add the nodes satisfying the condition to the bridge hub set B11:  Return B, IW, and IWBS

### 3.3. Comparison Methods and Evaluation Index

To verify the superiority and reliability of the proposed algorithm, this section describes the benchmark methods and defines the evaluation metrics adopted in the comparative experiments.

#### 3.3.1. Baseline Methods

To validate the effectiveness of the proposed IWBS-based key node mining algorithm, the following key node removal strategies are set as baselines for comparison:

Random Removal (RE): nodes are randomly selected for removal;

Degree Centrality Removal [4] (DE): nodes are removed in descending order of degree centrality;

Betweenness Centrality Removal [5] (BE): nodes are removed in descending order of betweenness centrality;

Hypernetwork Overlap Centrality Removal [27] (HO): removal based on the degree of node participation in hyperedge overlaps;

Interweaving Degree Removal (IW): nodes are removed in descending order of IW;

IWBS-based Key Node Removal (IWBS): nodes are removed in descending order of IWBS as proposed in this paper.

Among these, the first four methods are traditional or structure-heuristic approaches. The latter two methods incorporate the soft community partition results and service coupling features proposed in this paper, enabling more accurate identification of cross-community key nodes.

#### 3.3.2. Evaluation Metrics

To evaluate the removal effects, this paper adopts two perspectives: performance comparison analysis and cascading analysis. The general comparison metrics mainly include Service Efficiency (SE), Largest Connected Component (LCC), Service Disruption Rate (Disruption), Removal Cost (Cost), and Return on Investment (ROI). The cascading analysis metrics include Cascading Rounds (Rounds), Cascading Amplification Rate (CAR), Secondary Failure Ratio (SFR), Cross-Community Propagation Rate (CCPR), Community Spread Rate (CSR), Number of Reroute Events (Reroute Events), and Load Decline (LoadDecline). These metrics collectively provide a comprehensive assessment of the destructive effects of different removal strategies from multiple perspectives, including functional loss, structural degradation, propagation intensity, and removal cost. The specific formulas for the indicators are shown in Table 1:

## 4. Experimental Design and Result Analysis

This section conducts experimental validation and result analysis of the proposed key node mining algorithm from three perspectives: comparative experiments, in-depth analysis of cascading characteristics, and algorithm parameter analysis with performance evaluation. First, the advantages of the proposed method in key node identification and node removal effects are verified by comparing it with traditional or structure-heuristic methods. Second, based on a cascading removal model with a rerouting mechanism [38], the cascading removal characteristics and cross-community propagation behaviors induced by different methods are further analyzed to reveal the formation mechanisms of propagation-induced damage. Finally, the algorithm parameters are analyzed, the algorithm performance is evaluated, and targeted protection recommendations are provided based on case studies.

### 4.1. Experimental Setup

#### 4.1.1. Simulation Parameter Settings

In this section, the basic parameters are set for two parts: the physical network and the service network. The physical network is a multi-layer network topology consisting of a terrestrial user network and a satellite network at a given time. Several communication nodes, observation nodes, receiving nodes, and navigation nodes are generated according to population density and land–sea ratio. The seed satellites of the LEO satellite constellation adopt the orbital altitude of most LEO satellites (e.g., the OneWeb constellation) and employ the Walker constellation to establish the LEO satellite constellation. Meanwhile, a MEO constellation and a GEO constellation are also established, where the GEO constellation consists of a Walker constellation at the altitude of a geostationary orbit satellite with a uniform orbital inclination of 55°. The basic parameters of the specific constellations are shown in Table 2.

The parameters of the ground nodes are determined, and the numbers of communication, navigation, and remote sensing services are set in proportion to the basic constellation parameters, as shown in Table 3:

#### 4.1.2. Experimental Parameter Settings

The main parameters adopted in the experiments of this paper are as follows: the number of latent communities in the hypernetwork soft community partition is set to K=7; the entropy threshold is set to θ=0.6; and the bridge node screening ratio is set to 0.2. The parameters are set according to the classical cascading failure model [39] and the engineering constraints of the satellite network. Specifically, the capacity gain coefficient of 1.5 falls within the commonly used range of 1.2–2.0, balancing observable overload propagation with network stability. A load overflow coefficient of 0.25 accounts for the limited link margin and high inter-regional delay in satellite networks, where only a small fraction of failed loads can be successfully redistributed. The cross-community rerouting gain factor of 1.5 quantifies the extra cost of inter-community paths—i.e., more hops and longer delays—following the common practice in multilayer network models. A minimum capacity lower bound of 0.05 prevents numerical instability and has a negligible effect on the simulation results. Threshold parameters in metrics such as Service Efficiency and Service Disruption Rate are uniformly set according to service semantics and network structural characteristics. All experiments are repeated under the same random seed to ensure comparability and stability of the results.

### 4.2. Algorithm Validation and Comparative Experiments

#### 4.2.1. Parameter Settings

In the experiments, the removal ratio is set to multiple discrete levels, ranging from 5% to 30% with increments of 5% (i.e., 5%, 10%, 15%, 20%, 25%, and 30%). For RE, to reduce the fluctuation caused by randomness, the procedure is repeated with 30 independent samplings, and the results are averaged. For the other algorithms, the top-ranked nodes are selected as the removal targets in descending order of their respective rankings.

#### 4.2.2. Result Analysis

**Figure 4:**
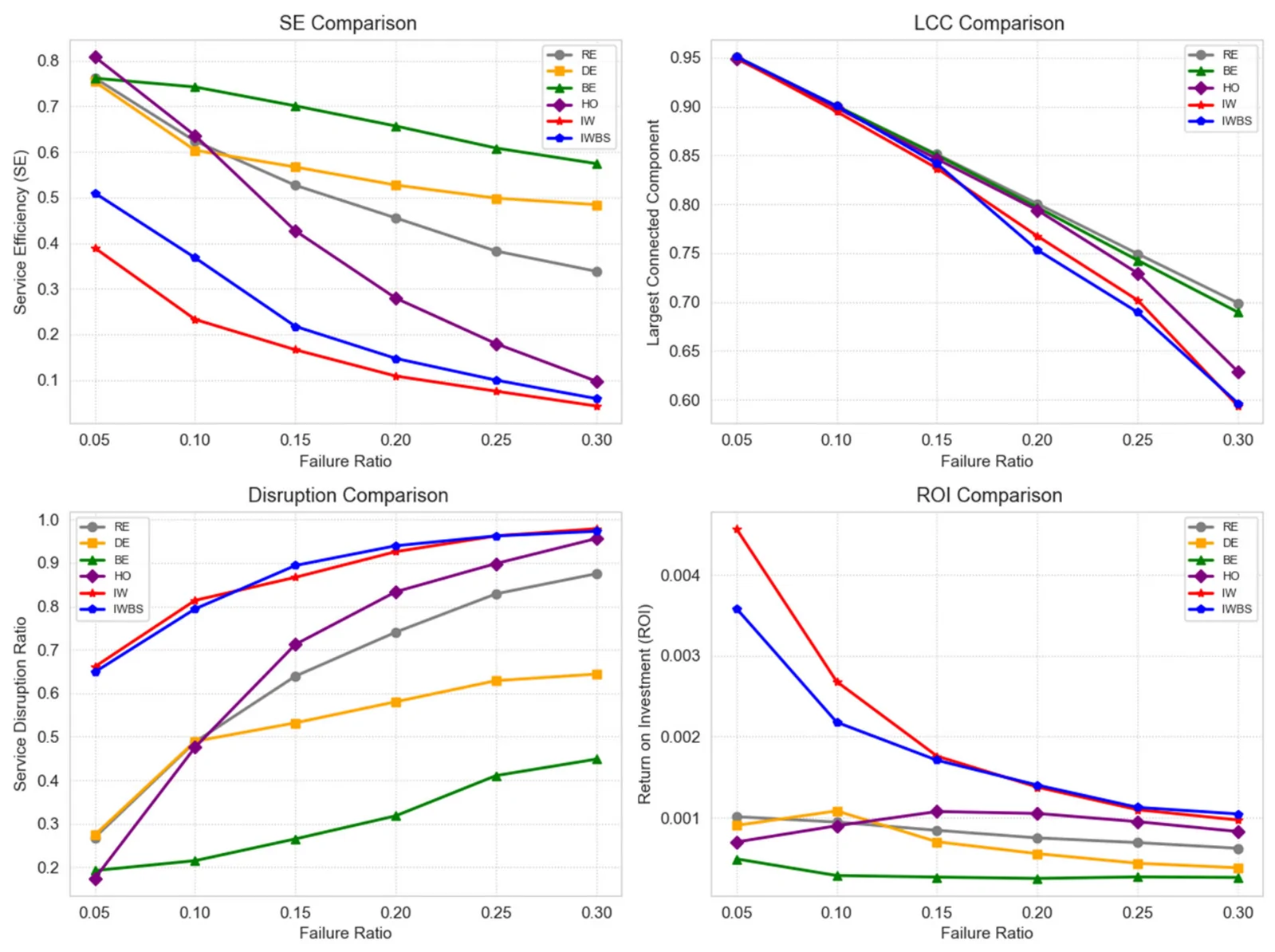
Comparison of Performance Metrics.

The experimental results show that as the removal ratio increases, all algorithms cause a decline in Service Efficiency (SE), a reduction in the Largest Connected Component (LCC), and an increase in the service disruption rate. However, the destructive effects differ significantly among the algorithms, as shown in Figure 4.

A detailed analysis is presented as follows:

(1) Service Efficiency (SE): IW and IWBS significantly outperform the other four strategies at all removal ratios. At a removal ratio of 5%, the SE of IW and IWBS drops to 0.389 and 0.510, respectively, while the SE of RE, DE, BE, and HO are 0.762, 0.754, 0.754, and 0.808, respectively. When the removal ratio increases to 30%, the SE of IW and IWBS further decreases to 0.043 and 0.059, respectively, whereas RE remains at 0.338—a difference of approximately six times. This phenomenon indicates that nodes with high IW or IWBS carry most of the service load in the network, and their removal causes a direct and rapid destruction of service efficiency.

(2) Largest Connected Component (LCC): IW and IWBS also cause the most severe damage to network connectivity. At a 30% removal ratio, the LCC of IW and IWBS drops to 0.594 and 0.596, respectively, while that of RE is 0.699. In contrast, DE and BE have a limited effect on LCC reduction. The reason is that after removing nodes with high degree or high betweenness centrality, the network can still maintain connectivity through redundant paths. This result demonstrates the limitation of conventional centrality metrics in hypernetwork service-coupled networks.

(3) Service Disruption Rate (Disruption): IW and IWBS cause more than 60% service disruption at a removal ratio of 5%, whereas BE causes only 25%. At a 30% removal ratio, both IW and IWBS cause a disruption rate exceeding 97%, essentially paralyzing the network. This indicates that removing IW or IWBS nodes can instantly trigger large-scale service disruption, and their destructive efficiency is much higher than that of metrics based on ordinary graph topology.

(4) Return on Investment (ROI): In the low removal ratio range (5–10%), IW has the highest ROI (0.00457 at 5%), followed by IWBS (0.00358), and BE has the lowest (0.00049). That is, the service efficiency loss per unit node removal cost of IW is approximately nine times that of BE. As the removal ratio increases to 30%, the ROI of IWBS (0.00105) is slightly higher than that of IW (0.00097), indicating that IWBS has higher marginal benefits under high-intensity node removal.

Based on the above performance metrics, IW and IWBS are significantly superior to traditional graph-theoretic methods in terms of destructive effects. Although DE and BE can identify nodes with more edges or more paths in the topological structure, their focus is limited to ordinary graph topology and they cannot fully capture the service coupling and cross-community propagation risks in hypernetworks. HO, to some extent, characterizes local overlapping features in hypernetworks but still struggles to accurately identify service-dependent key nodes due to the lack of a joint representation of service coupling.

### 4.3. In-Depth Analysis of Cascading Characteristics

According to the results in Section 4.1, the proposed IWBS algorithm performs comparably to the IW algorithm on conventional performance metrics, and both exhibit clear advantages over traditional graph-theoretic methods and hypernetwork structural metrics. To further analyze the intrinsic propagation mechanisms induced by the removal of IWBS- and IW-identified nodes, demonstrate the superiority of IWBS in macroscopic structural mining, and clarify the distinct application scenarios of the two algorithms, this section adopts CAR, SFR, CCPR, and CSR to reveal the underlying mechanisms of removal propagation following node removal.

#### 4.3.1. Comparison of Cascading Characteristics

**Figure 5:**
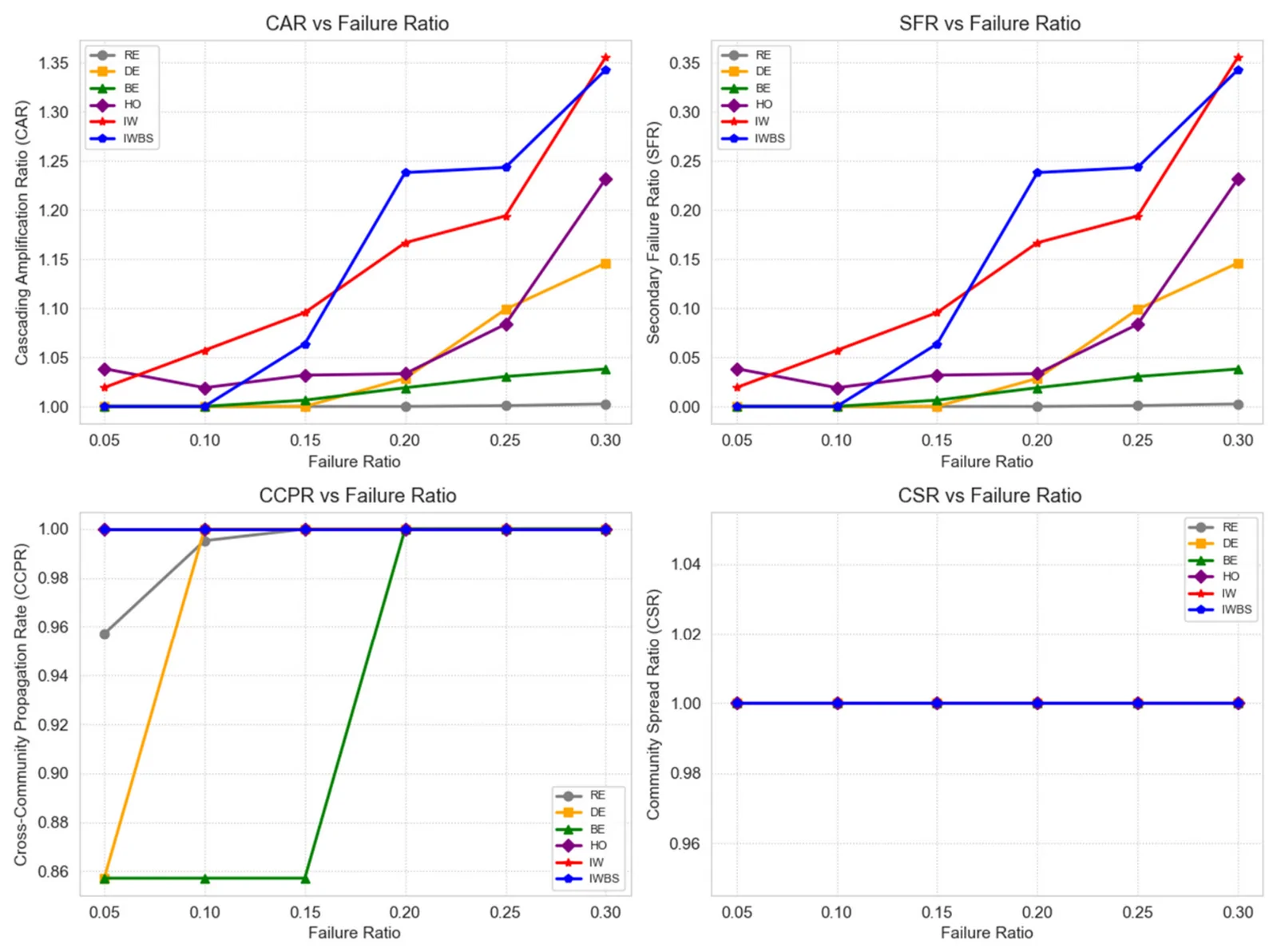
Comparison of Cascading Characteristics.

As shown in Figure 5, at a removal intensity of 30%, the CAR of IWBS reaches 1.34, while that of IW is 1.36, and that of RE is only 1.00, inducing almost no cascading effect. This indicates that the removal of critical hubs indeed triggers a wider range of chain reactions. The Secondary Failure Ratio (SFR) more directly quantifies the additional loss caused by cascading propagation. At 30% intensity, the SFR of IWBS is 0.34, meaning that for every 100 nodes removed, an additional 34 nodes fail due to cascading effects.

Comparing IWBS and IW directly, it can be observed that when the number of removed nodes is small, IW exhibits a stronger cascading effect. However, as the removal number increases, IWBS gradually surpasses IW. Particularly when the removal ratio exceeds 20%, the cascading rounds of IWBS are significantly longer, reaching up to 8 rounds, while IW stabilizes at 4–5 rounds. This indicates a fundamental difference in the damage patterns of the two algorithms.

This phenomenon suggests that IWBS is not limited to reducing the number of local nodes; rather, it possesses a remarkable removal propagation amplification property. Compared with IW, IWBS does not necessarily cause the strongest instantaneous impact first, but by cutting off bridge channels between communities, it forces traffic to reorganize and redistribute over a wider area, thereby triggering longer-round and higher-amplitude cascading removals. Therefore, if one relies solely on traditional metrics such as SE or LCC, the actual risk of bridge nodes may be underestimated, whereas CAR, SFR, CCPR, and CSR can more accurately reflect such propagation-dominated damage behaviors.

#### 4.3.2. Mechanism Analysis of Removal Patterns

To comprehensively compare the network damaging effects of IW and IWBS under different removal intensities, this subsection quantitatively analyzes three dimensions: service efficiency loss (SE drop), service disruption rate (Disruption), and propagation metrics (CAR and SFR).

1. Evolution of Service Efficiency Loss and Service Disruption Rate

**Figure 6:**
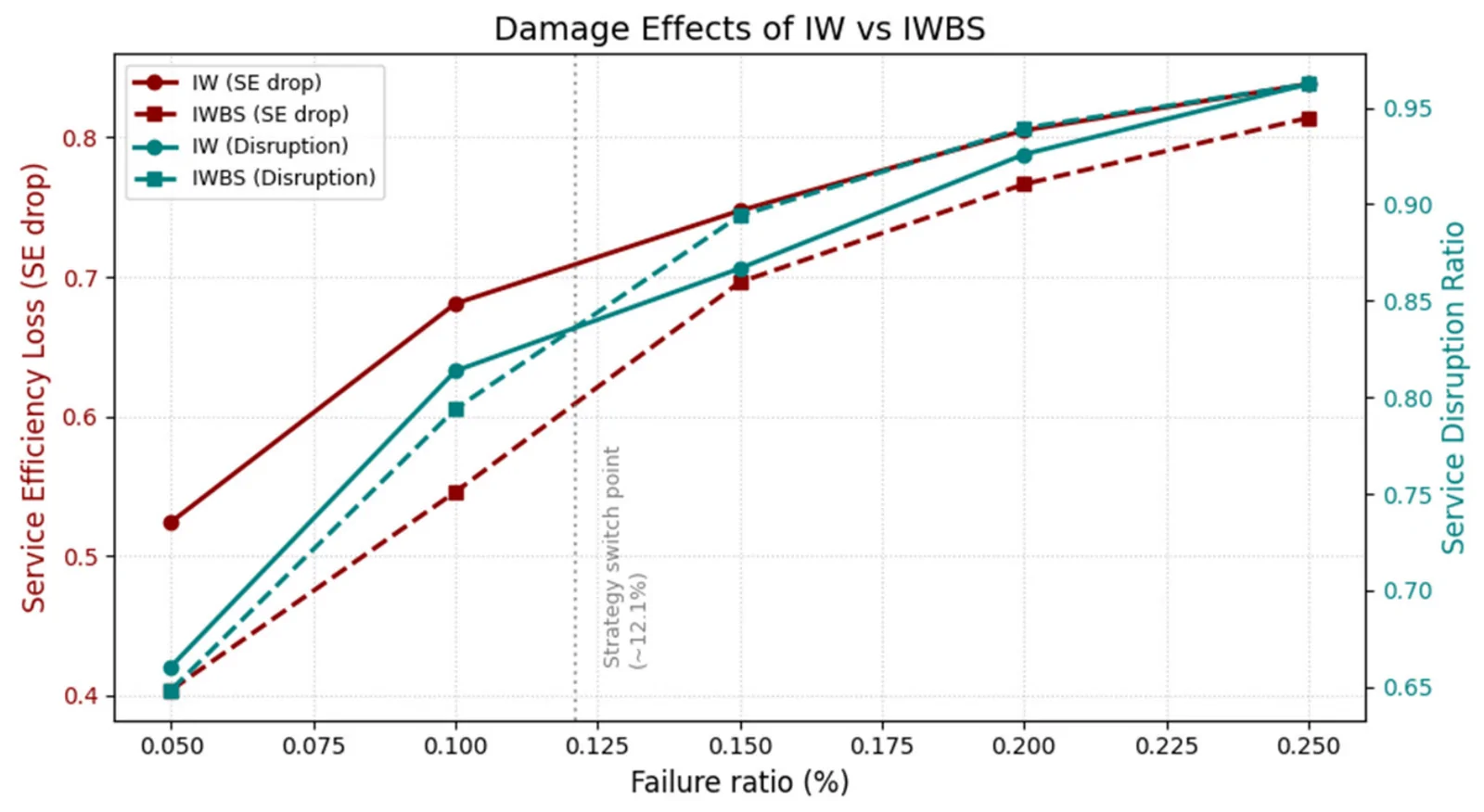
Comparison of Failure Characteristics between IW and IWBS.

Figure 6 shows the variation curves of service efficiency loss and service disruption rate with removal intensity under the two removal modes. In terms of service efficiency loss, IW consistently outperforms IWBS in terms of destructive effect. At low removal intensity (5%), the SE drop of IW is 0.524, while that of IWBS is only 0.404. As the removal intensity increases to 25%, the SE drop of IW reaches 0.838, and that of IWBS reaches 0.814. This indicates that directly removing high-IW nodes can more effectively reduce network service execution efficiency, and this advantage is maintained across the entire intensity range.

Regarding the service disruption rate, the difference between the two is small at low intensities (5–10%), with IW slightly higher than IWBS. When the removal intensity reaches 15%, the disruption rate of IWBS (0.894) overtakes that of IW (0.867) and maintains a slight lead at 20–25% intensity. By linear interpolation, the intersection point of the two disruption rate curves is located at a removal intensity of approximately 12.1%. This point can be defined as the strategy switching threshold: below this threshold, IW causes higher direct service disruption; above this threshold, the disruption range caused by IWBS due to cascading diffusion gradually exceeds that of IW.

2. Comparative Analysis of Propagation Metrics

**Figure 7:**
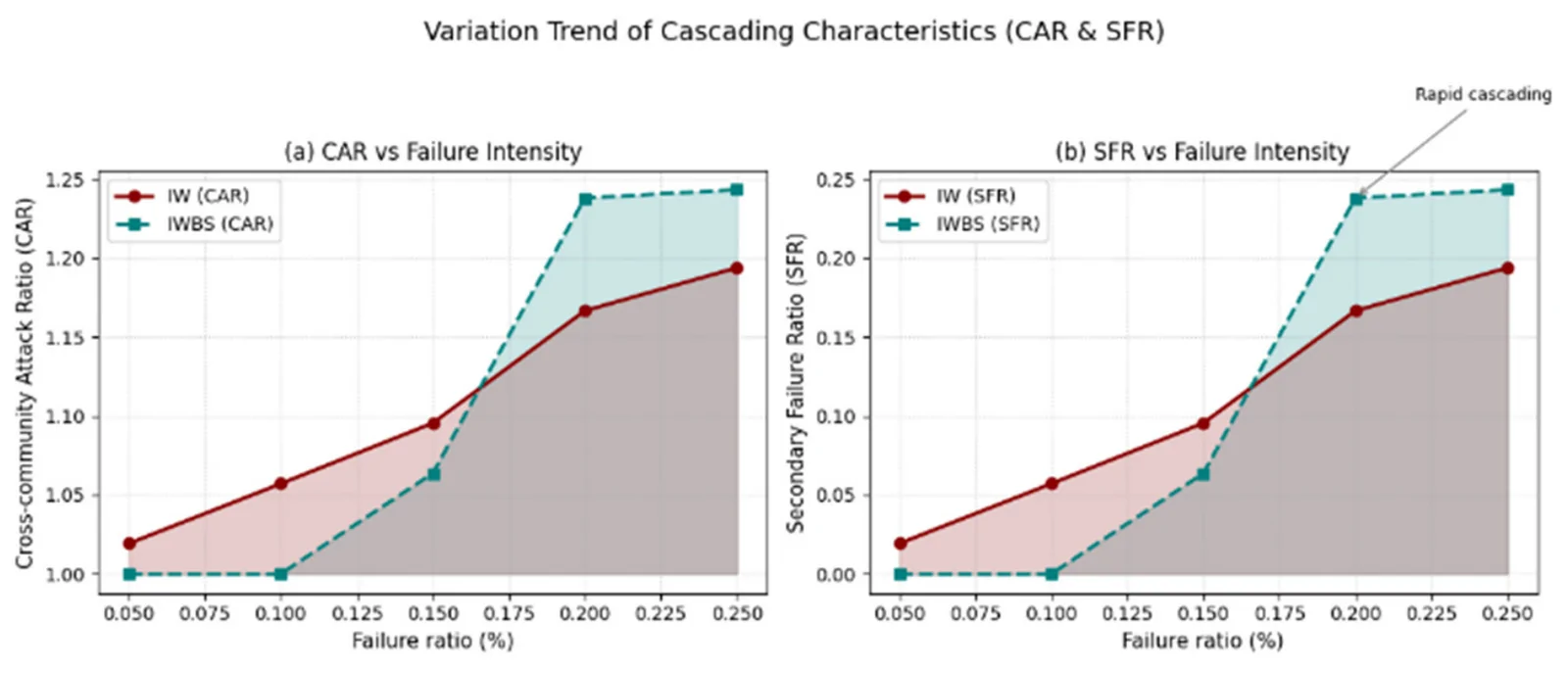
Comparison of Propagation Characteristics between IW and IWBS.

Figure 7 shows the variations in the Cascading Amplification Rate (CAR) and the Secondary Failure Ratio (SFR) with removal intensity. For CAR, the value of IW remains slightly above 1 (ranging from 1.019 to 1.194), while that of IWBS stays at 1 for low intensities (≤10%) and then rapidly increases to 1.243 after the intensity exceeds 15%. This indicates that the removal effect of IW involves cross-community propagation from the very beginning, whereas IWBS only significantly triggers inter-community diffusion after exceeding a threshold.

The difference in SFR is even more pronounced. The SFR of IW grows slowly from 0.019 to 0.194, while that of IWBS is 0 at 5–10% intensity, jumps to 0.064 at 15%, and reaches 0.238 and 0.243 at 20% and 25% intensity, respectively, exhibiting an approximately exponential growth pattern. Combined with the cascading rounds (8 rounds for IWBS at 25% intensity, far exceeding the 4 rounds of IW), it can be confirmed that IWBS is inherently a propagation-type removal strategy, whose destructive effect amplifies nonlinearly with increasing removal intensity; IW, on the other hand, is a direct-type removal strategy with limited propagation capability.

**Figure 8:**
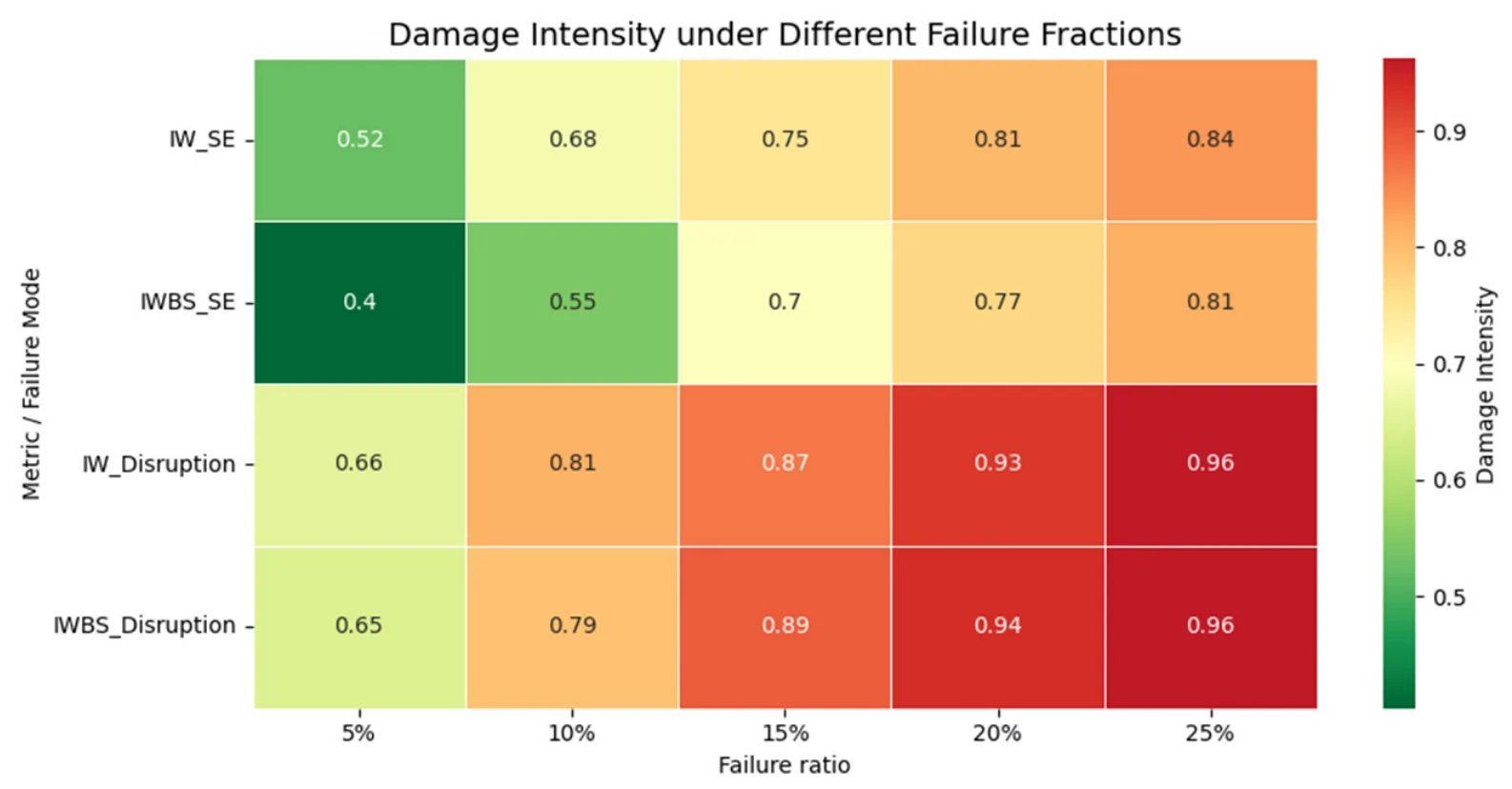
Damage Intensity Heatmap.

3. Damage Intensity Heatmap Analysis:

The heatmap in Figure 8 unifies the above metrics into a common color-gradient matrix. It can be intuitively observed that for IW, both SE drop and disruption rate exhibit a deepening red trend with increasing removal intensity, and the high-value region (≥0.8) covers the 20–25% intensity range. For IWBS, the SE drop is lower than that of IW, but its disruption rate is comparable to that of IW (0.96) at high intensities. Meanwhile, the high-value regions (dark red) of SFR and CAR are clearly concentrated in the 20–25% intensity range, verifying their propagation characteristic of “latency at low intensity and outbreak at high intensity.”

In summary, by comparing the two removal modes, IW and IWBS, the following conclusions can be drawn:

(1) Fundamental difference in removal modes:

IW is a direct-damage type: removal of high-interweaving-degree nodes immediately causes significant service efficiency loss and local service disruption, with weak propagation.

IWBS is a propagation-damage type: removal of bridge nodes causes little direct damage initially, but triggers cascading overload and cross-community diffusion. Under high removal intensity, its damage scope and depth surpass those of IW.

(2) Strategy switching threshold:

The service disruption rate curves cross at a removal intensity of approximately 12.1%. When the expected removal intensity is below this threshold, IW has higher disruption efficiency; above this threshold, IWBS poses a greater long-term threat. Therefore:

For short-term rapid service paralysis (e.g., battlefield temporary suppression, emergency jamming): IW is preferred, achieving an SE drop exceeding 0.5 and a service disruption rate above 0.66 at 5–10% intensity.

For long-term systematic degradation (e.g., strategic resource consumption, infrastructure suppression): IWBS should be selected, ensuring a removal intensity ≥ 15% to leverage its exponential propagation characteristics for full-network functional degradation.

(3) Defense strategies:

Against IW: redundantly deploy high-interweaving-degree nodes and design fast reconfiguration mechanisms.

Against IWBS: strengthen protection of cross-community bridging links, deploy elastic traffic routing and community isolation strategies to block cascading diffusion.

### 4.4. Algorithm Ablation Analysis and Performance Evaluation

#### 4.4.1. Parameter Sensitivity Analysis

**Figure 9:**
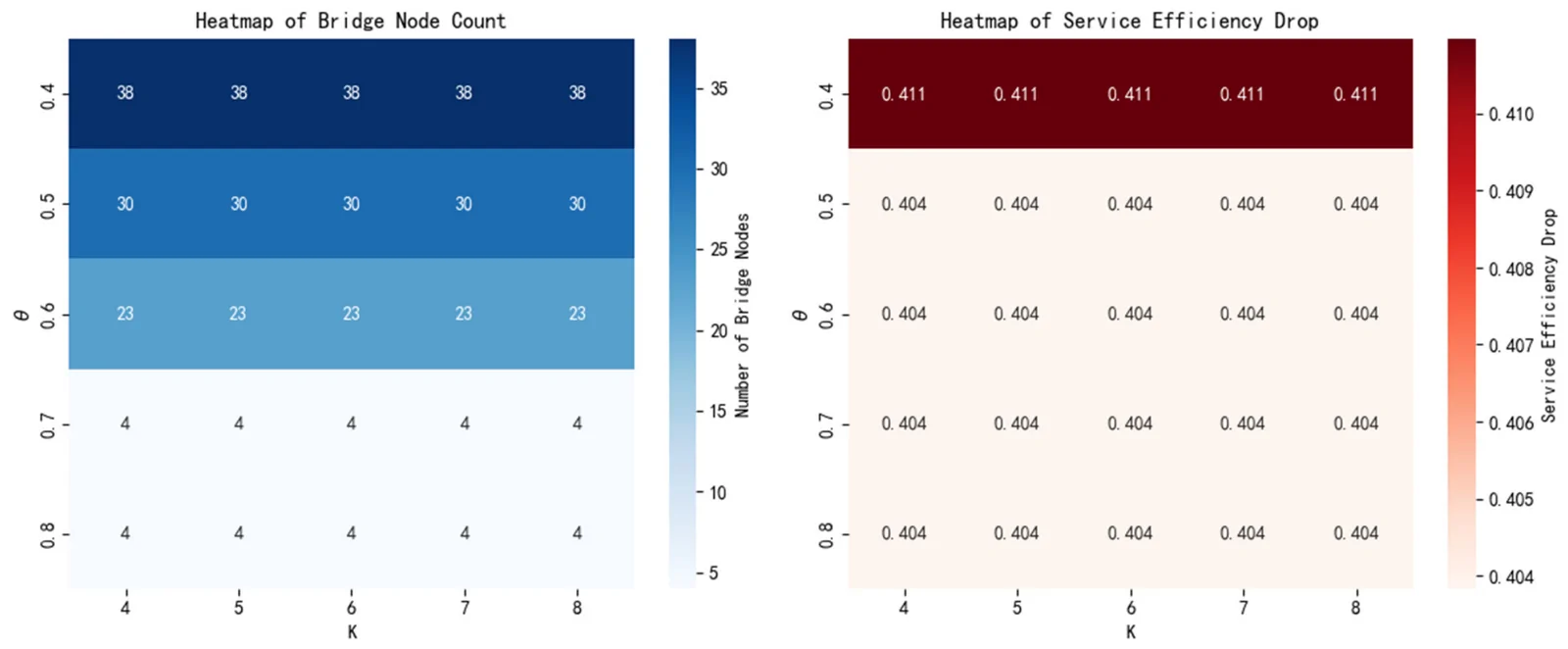
Parameter Sensitivity Analysis.

To evaluate the influence of community partition parameters on the stability of the IWBS algorithm, we fix the removal ratio at f=0.05 and traverse over θ∈{0.4,0.5,0.6,0.7,0.8} and K∈{4,5,6,7,8}, recording the number of bridge nodes and the service efficiency loss. The results are shown in Figure 9.

The number of bridge nodes decreases exponentially as θ increases: when θ=0.4 the average is 38; at θ=0.5, 30; at θ=0.6, 23; and when θ≥0.7, it drops sharply to 4. Under different values of K, the difference in the number of bridge nodes corresponding to the same θ does not exceed 2. This indicates that θ is the core parameter for bridge node screening, while the influence of K is negligible.

The service efficiency loss remains constant at 0.40383 when θ≥0.5, and is only slightly higher at 0.41097 when θ=0.4, a relative difference of 1.8%. This demonstrates that the IWBS algorithm has strong robustness to parameter selection: even when the threshold varies from 0.5 to 0.8, or the number of communities is arbitrarily chosen between 4 and 8, the removal efficiency remains almost unchanged. This stability stems from the unsupervised nature of hypergraph soft community partition: as long as the degree of community overlap is moderate, the critical bridge nodes can be uniquely identified, and the service coupling parameters only play a fine-tuning role.

In summary, the recommended parameter interval is θ∈[0.5,0.6] and K∈[5,7]. Within this interval, the algorithm performance is stable and does not require fine parameter tuning, facilitating engineering deployment.

#### 4.4.2. Ablation Analysis

To quantify the independent contributions of the two core modules—overlapping community partition and cross-community propagation evaluation—to the IWBS algorithm, five model variants are designed and compared with random removal:

Full: the complete IWBS algorithm, including soft community partition, overlap degree features, and cross-community propagation evaluation.

w/o Overlap: removes the overlap degree feature (i.e., no longer considers whether a node is located in overlapping areas of multiple communities), retaining only community membership.

w/o Cross: removes the cross-community propagation evaluation feature (i.e., considers only the importance of a node within its own community, ignoring the bridging role between communities).

w/o Soft-Comm: removes soft community partition, degenerating to pure Interweaving Degree (IW) ranking without distinguishing community overlaps.

w/o Bridge Enhance: removes the bridge enhancement term. Since its result is identical to that of w/o Soft-Comm, they are analyzed together.

This experiment is conducted at a removal intensity of f=0.25 (i.e., removing 25% of the nodes in the network). The evaluation metrics cover seven dimensions: service efficiency loss (SE drop), service disruption rate (Disruption), cascading rounds (Rounds), cascading amplification rate (CAR), secondary failure ratio (SFR), return on investment (ROI), and cost efficiency (Cost Efficiency = 1/AttackTotalCost). All metrics are normalized to the interval $\left[0,1\right]$, where a larger value indicates better performance in that dimension.

**Figure 10:**
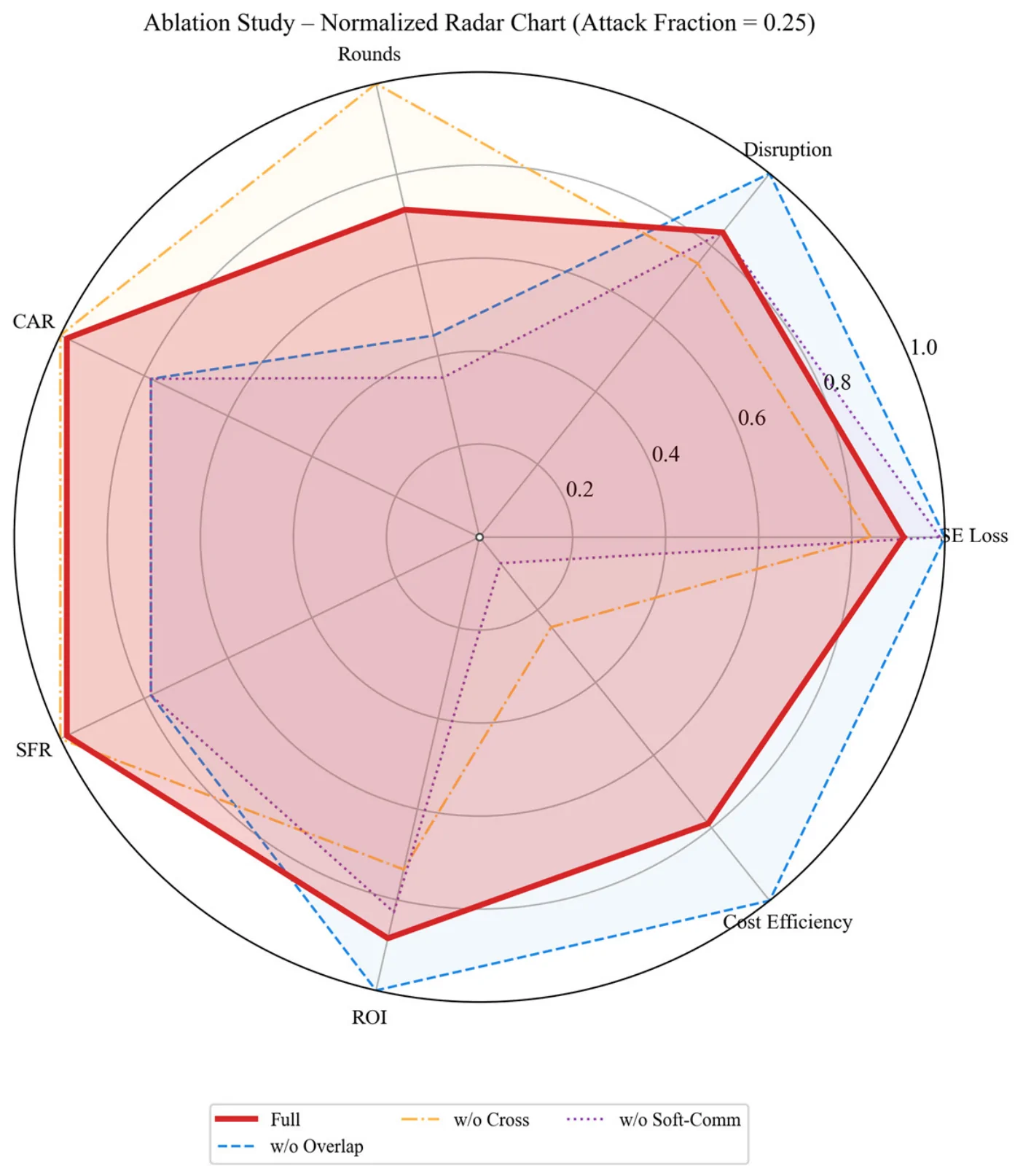
Ablation Analysis.

As shown in Figure 10, the Full model covers the largest area, with optimal cost efficiency and ROI, while the other metrics are at an intermediate to high level, indicating that it achieves the best balance between direct damage and deep cascading propagation, combining the lowest cost with the highest return. w/o Soft-Comm achieves the highest scores in SE drop and disruption rate, but its cost efficiency and ROI are low, and its cascading capability is inferior to that of Full, making it suitable for low-intensity short-term suppression but with poor long-term benefits. w/o Overlap has an SE drop close to that of w/o Soft-Comm, but its cascading depth and cost efficiency are lower than those of Full, indicating that removing the overlap feature expands the removal surface but sacrifices propagation sustainability and economic efficiency. w/o Cross scores low across all metrics, with particularly poor cascading and cost efficiency, confirming that the cross-community feature is the core mechanism for triggering long-chain cascading; its absence leads to overall algorithm degradation. Random (RE) covers the smallest area and has the worst performance across all metrics, demonstrating the necessity of targeted removal.

In summary, the Full model achieves the best overall performance. Overlapping community partition ensures the accuracy of bridge node identification, while cross-community propagation evaluation endows the algorithm with long-chain cascading capability; both are indispensable. Pure Interweaving Degree removal has an advantage only in instantaneous suppression. Removing the overlap feature weakens propagation sustainability; removing the cross-community feature leads to overall algorithm degradation. Therefore, overlapping community partition and cross-community propagation evaluation are both core components of the IWBS algorithm; the absence of either significantly degrades performance or alters the damage pattern.

#### 4.4.3. Computational Scalability

**Figure 11:**
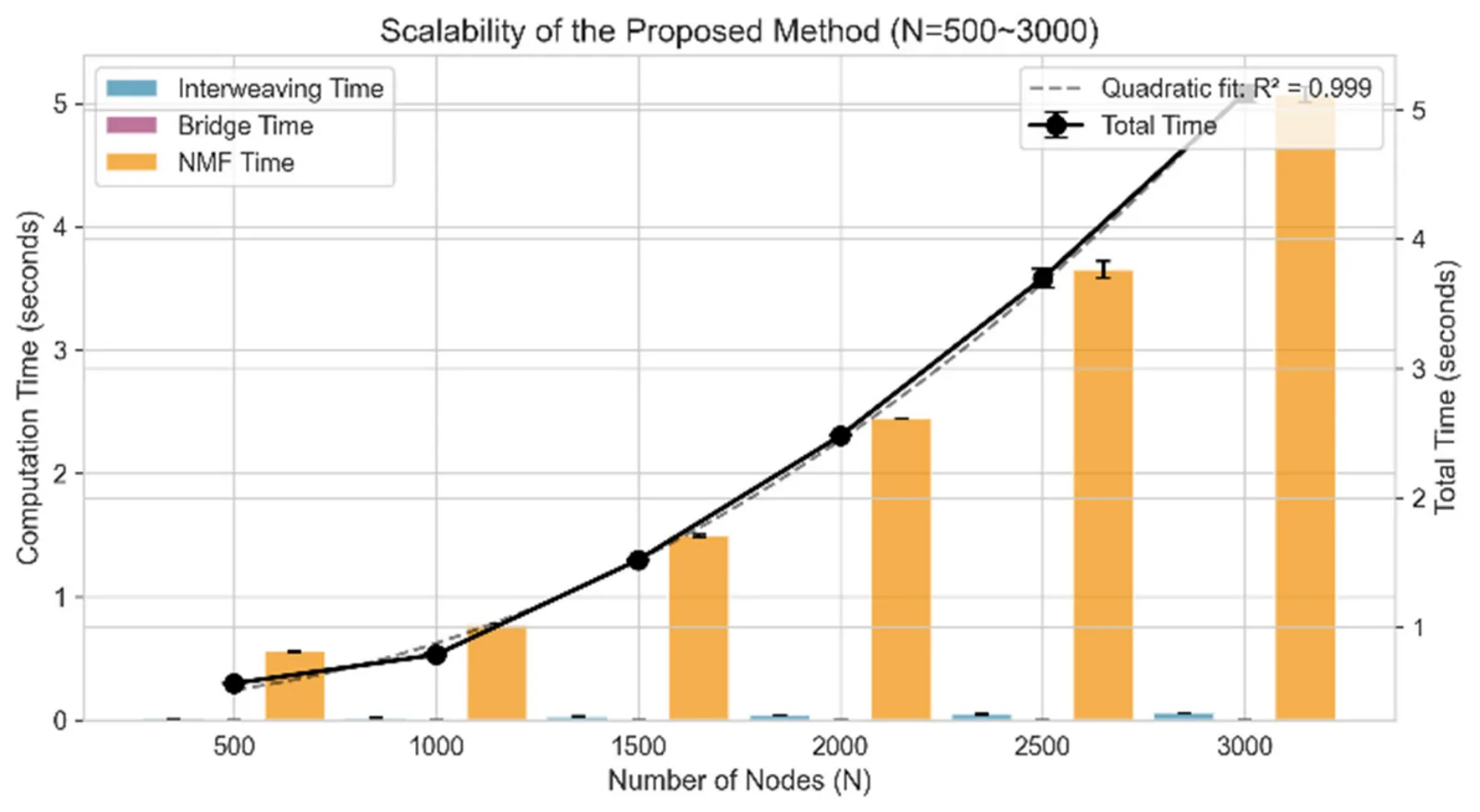
Scalability of the Proposed Method.

In large-scale service-coupled networks, the computational efficiency of an algorithm is a core metric determining its engineering feasibility. To evaluate the real-time performance of the IWBS algorithm under different network scales, we tested the computation time of each component when the number of nodes is N={100,200,400,600,800,1000}. For each scale, the procedure was repeated three times, and the mean and standard deviation were calculated. To intuitively compare time components differing by several orders of magnitude, Figure 11 uses a dual-axis chart: the left axis shows a logarithmic-scale bar chart (for Interweaving Degree calculation, bridge node identification, and NMF decomposition), and the right axis shows a linear-scale line chart of the total computation time along with a linear regression fit line.

The NMF decomposition time dominates the total computation time, accounting for over 97%. It is approximately 0.55 s when N = 500 and grows to about 5.1 s when N = 3000, exhibiting a nearly linear increase (fitted slope of approximately 0.0017 s per node). The computation time for the interweaving degree increases gently, from 0.010 s (at N = 500) to 0.055 s (at N = 3000). The bridge node identification time remains below 0.0001 s and is negligible.

Although the NMF step accounts for the majority of the total computation time, it only needs to be recomputed offline when the network structure changes (e.g., service updates or node joining/leaving). In contrast, the online node importance evaluation always remains at the level of tens of milliseconds, fully meeting the requirements for routine and real-time monitoring. For even larger-scale scenarios ($N>10^{4}$), incremental NMF or community approximation techniques can be adopted to further reduce the offline overhead. Therefore, the IWBS algorithm exhibits good scalability and can support the analysis services of medium- to large-scale service-coupled networks.

#### 4.4.4. Community Partition Quality Validation

**Figure 12:**
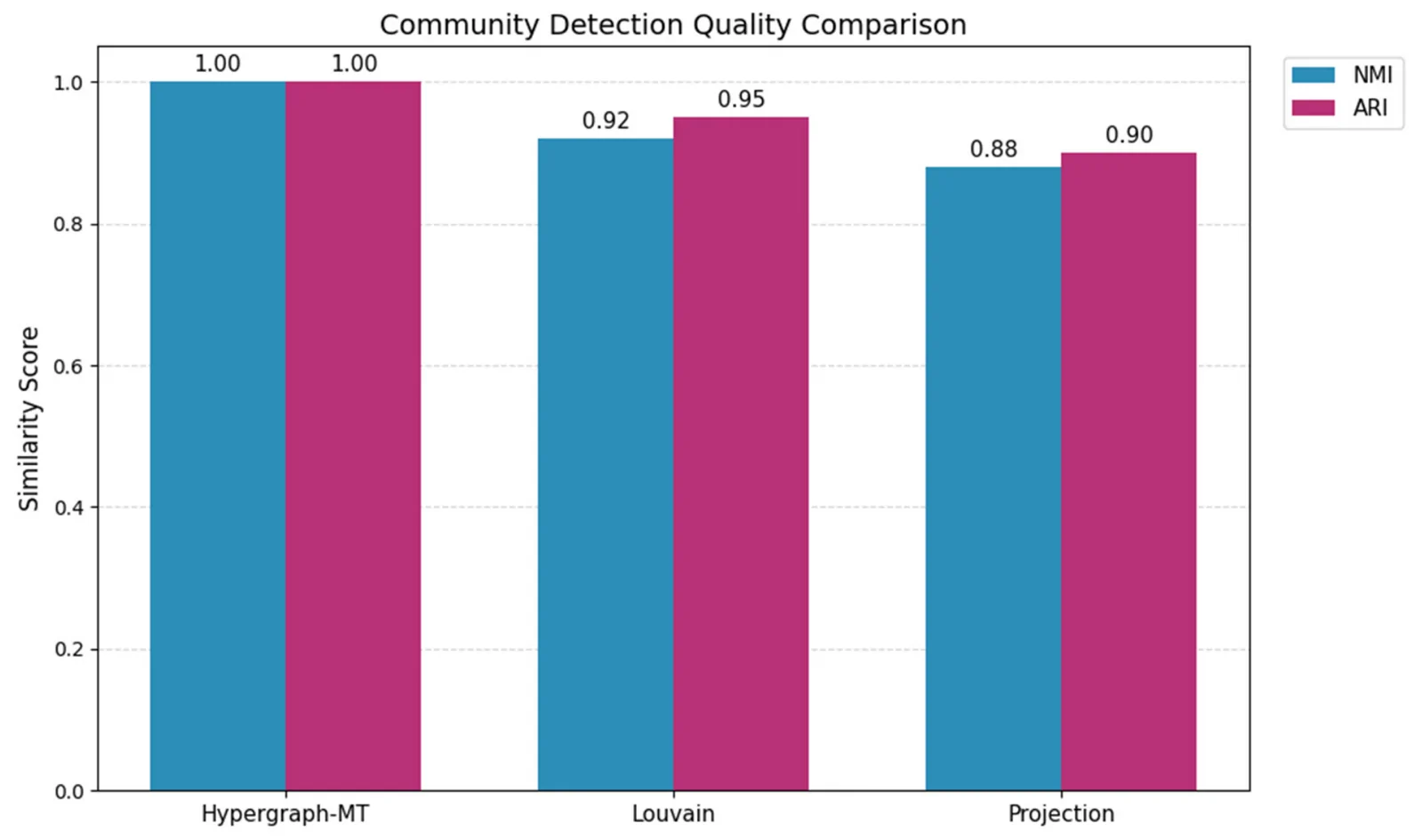
Comparison of Community Partition Algorithms.

The identification of high-IWBS bridge nodes relies on accurate partition of overlapping communities. To validate the effectiveness of the hypernetwork multi-service community partition method (Hypergraph-MT) adopted in this paper, we compare it with two mainstream baseline methods: ① Louvain [15] and ② Projection [16]. The evaluation metrics are Normalized Mutual Information (NMI) and Adjusted Rand Index (ARI), both ranging in $\left[0,1\right]$, where higher values indicate better agreement between the partition results and the ground-truth community structure. The results are shown in Figure 12.

Among them, Hypergraph-MT achieves an NMI of 1.00 and an ARI of 1.00, perfectly recovering the preset community structure. Louvain yields NMI = 0.92 and ARI = 0.95, indicating that ordinary graph models lose the high-order multi-node association information contained in hyperedges. Projection further degrades to NMI = 0.88 and ARI = 0.90, demonstrating that simple hypernetwork projection may introduce spurious edges or miss true community boundaries.

Therefore, this method can simultaneously capture the overlapping roles of nodes in multiple services, providing an accurate structural foundation for subsequent bridge node mining.

#### 4.4.5. Node Risk Level Assessment and Protection Recommendations

**Figure 13:**
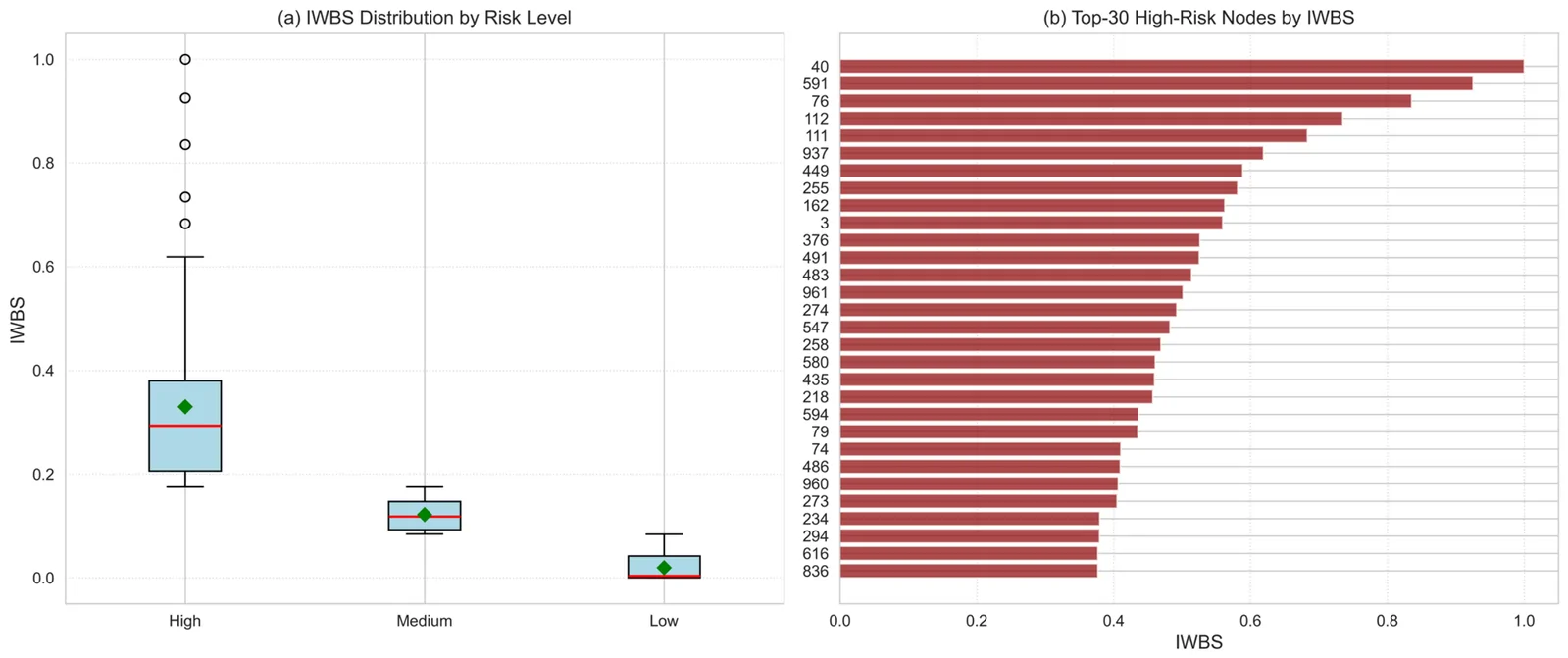
Statistical Characteristics of Risk Levels. (**a**) IWBS Distribution by Risk Level. (**b**) Top-30 High-Risk Nodes by IWBS.

**Figure 14:**
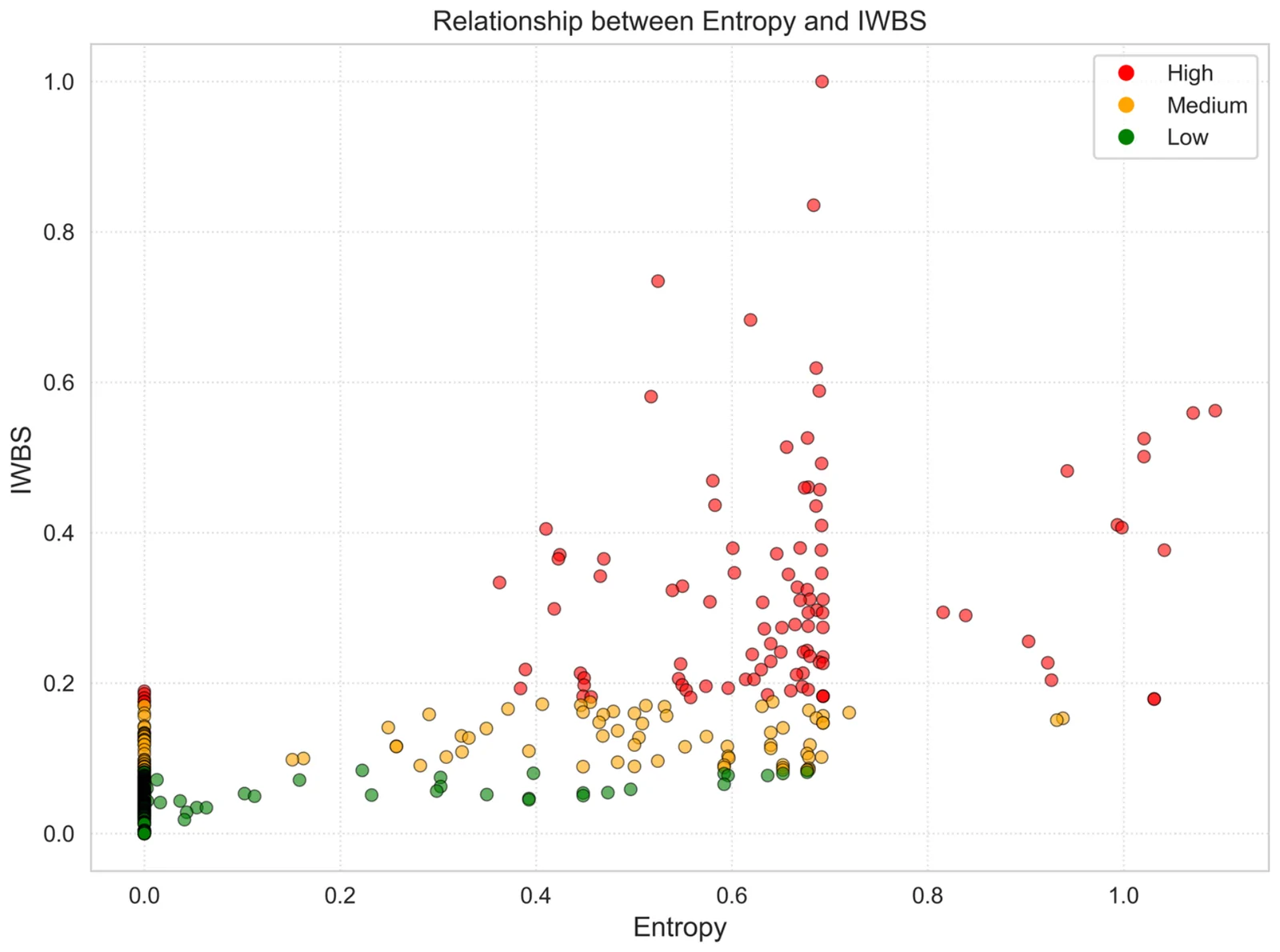
Distribution of Key Nodes.

Based on the IWBS score, entropy value, and community participation degree, we classify network nodes into three risk levels—high, medium, and low—and propose differentiated engineering protection recommendations for each level. Figure 13 and Figure 14 show the statistical characteristics of the risk levels and the distribution of key nodes.

1. Distribution of IWBS scores

The boxplot shows that the median IWBS score of high-risk nodes is approximately 0.30, significantly higher than that of medium-risk nodes and low-risk nodes. Moreover, the interquartile range (IQR) of high-risk nodes is small, indicating that their scores are concentrated in the upper quartile, demonstrating the excellent discriminative ability of the IWBS score.

2. Ranking of high-risk nodes

The IWBS scores of the top 30 high-risk satellite nodes range from 0.999 to 0.18. Among them, node 40 has an IWBS score close to 1.0, an entropy value of about 0.69, and a community participation degree of about 0.50, indicating that it simultaneously carries extremely high service loads and lies in overlapping areas of multiple communities. For such nodes, priority protection strategies such as redundant deployment, backup routing, and capacity reservation are recommended. For medium-risk nodes, dynamic load balancing and periodic health checks are suggested; low-risk nodes only require routine monitoring.

3. Relationship between IWBS and entropy

The scatter plot reveals that high-risk nodes typically have moderate entropy values and extremely high IWBS scores, whereas low-risk nodes have widely distributed entropy but very low IWBS scores. This phenomenon indicates that a single structural entropy metric is insufficient to measure node threat: too low entropy implies that the node belongs to only a single community, while too high entropy indicates that the node belongs to too many communities, in which case the actual load is dispersed. Only moderate entropy combined with high service coupling can form truly destructive bridge nodes.

## 5. Conclusions and Future Work

This paper addresses the problem of key node mining in SINs under multi-service collaborative scenarios and proposes a key node mining method based on soft community partition and hypergraph interweaving degree. To overcome the limitations of traditional graph models in representing multi-node cooperative relationships and the inability of conventional centrality metrics to reveal cross-community propagation risks, this paper systematically investigates network modeling, community partition, and key node identification, making the following contributions.

First, this paper uniformly abstracts communication services, remote sensing services, and navigation services in satellite networks into a hypernetwork structure, enabling multi-node cooperative relationships to be modeled and analyzed within a unified framework. Compared with ordinary graphs, the hypernetwork can more realistically describe the high-order service coupling relationships existing in satellite networks, providing a more realistic structural foundation for subsequent key node identification.

Second, this paper adopts a KL-NMF-based hypernetwork soft partition method to perform soft community partition on the hypernetwork, obtaining probabilistic memberships of nodes to multiple communities. This method preserves the overlapping and boundary attributes of nodes, can better characterize the fuzzy community structure in satellite hypernetworks, and provides fundamental support for the construction of entropy, participation coefficient, and bridge hubs.

Third, this paper defines the Interweaving Degree (IW) metric to measure the coupling strength of nodes in communication, remote sensing, and navigation services. On this basis, it further integrates the community participation coefficient and community entropy to propose the IWBS-based key node mining algorithm. Experimental results show that the IWBS-based algorithm can more accurately identify key nodes that possess both high service coupling and high cross-community propagation capability, and their removal is more likely to trigger large-scale cascading diffusion.

Finally, through comparative algorithm verification, in-depth analysis of cascading characteristics, algorithm parameter analysis, and performance evaluation, the effectiveness, stability, and rationality of the proposed method are validated. Overall, the key node mining method presented in this paper can not only identify key nodes in SINs under multi-service collaborative scenarios but also explain the propagation mechanisms triggered by the removal of key nodes, providing theoretical support and methodological guidance for the defense management and construction planning of SINs.

Although this paper has made progress in key node mining algorithms for SINs, there remain several avenues for further exploration. First, future research can further investigate time-varying hypernetwork modeling methods for dynamic satellite networks, integrating node position changes, link availability variations, and service scheduling changes into a unified dynamic structural analysis framework, thereby enhancing the model’s adaptability to real operational environments. Second, more efficient algorithms for community partition and key node identification can be explored, such as combining graph neural networks, tensor decomposition, or deep representation learning methods, to improve computational efficiency and identification accuracy on large-scale networks.

In summary, this paper provides a hypernetwork-based structured modeling approach for SIN topology analysis and a key node mining algorithm that incorporates both community attributes and multi-service attributes. Future extensions in three directions—dynamic scenarios, complex constraints, and defense mechanisms—are expected to provide stronger theoretical support for the security and stability design of next-generation SINs.

## Figures and Tables

**Table 1:** Metric Definitions.

Type	Metric	Formula	Trend
Performance	SE	$SE(SIN)=\frac{\sum_{e_{i}^{H}\in E^{H}}\omega_{i}\times SE(e_{i}^{H})}{\sum_{j=1}^{\left|E^{H}\right|}\omega_{j}}$	−
	LCC	$LCC=\frac{\left|C_{\max}\right|}{N}$	−
	Disruption	$Dis=\frac{E_{total}-E_{alive}}{E_{total}}$	+
	Cost	$\mathrm{Cos}t=C_{base}\cdot (1+\lambda \frac{\bar{d}}{d_{0}})$	-
	ROI	$ROI=\frac{△SE}{\mathrm{Cos}t}$	+
Cascading	CAR	$CAR=\frac{N_{final}}{N_{initial}}$	+
	SFR	$SFR=\frac{N_{final}-N_{initial}}{N_{initial}}$	+
	CCPR	$CCPR=\frac{C_{affected}}{C_{total}}$	+
	CSR	$CSR=\frac{C_{affected}}{C_{initial}}$	+

**Table 2:** Basic constellation parameters.

Type	Orbit Altitude (km)	Orbit Inclination (°)	Number of Orbital Planes	Satellites per Plane
LEO	1200	55	10	10
MEO	20,000	55	5	5
HEO	35,786	55	1	3

**Table 3:** Service parameters.

Service Type	Communication Service	Navigation Service	Remote Sensing Service	
Node type	Communication node	Navigation node	Observation point	Receiving point
Quantity/Scale	300	300	30	15

## Data Availability

The original contributions presented in the study are included in the article; further inquiries can be directed to the corresponding author.

## Abbreviations

The following abbreviations are used in this manuscript:

SINs	Spatial Information Networks
IW	Interweaving Degree
IWBS	Interweaving Degree-based Bridging Score
LEO	Low Earth Orbit
MEO	Medium Earth Orbit
HEO	High Earth Orbit
RE	Random Removal
DE	Degree Centrality Removal
BE	Betweenness Centrality Removal
HO	Hypernetwork Overlap Centrality Removal
NMF	Non-negative Matrix Factorization
PC	Participation Coefficient
ROI	Return on Investment
SE	Service Efficiency
TT&C	Tracking, Telemetry, and Control
KL-NMF	Kullback–Leibler Divergence-based Non-negative Matrix Factorization
CNR	Communication, Navigation, and Remote Sensing
CSR	Community Spread Rate
